# Photogenerated reactive oxygen species and hyperthermia by Cu_3_SnS_4_ nanoflakes for advanced photocatalytic and photothermal antibacterial therapy

**DOI:** 10.1186/s12951-022-01403-y

**Published:** 2022-04-20

**Authors:** Yangzi Yang, Chengwei Wang, Ning Wang, Jiaxin Li, Yingchun Zhu, Jiantao Zai, Jingke Fu, Yongqiang Hao

**Affiliations:** 1 Shanghai Key Laboratory of Orthopaedic Implant, Department of Orthopaedic Surgery, Shanghai Ninth People’s Hospital, Shanghai Jiao Tong University School of Medicine, Shanghai, 200011 China; 2 Clinical and Translational Research Center for 3D Printing Technology, Shanghai Ninth People’s Hospital, Shanghai Jiao Tong University School of Medicine, Shanghai, 200011 China; 3 Department of Orthopedics, The Second Affiliated Hospital of Harbin Medical University, Harbin, 150081 China; 4 Shanghai Electrochemical Energy Devices Research Center, School of Chemistry and Chemical Engineering and State Key Laboratory of Metal Matrix Composites, Shanghai Jiao Tong University, Shanghai, 200240 China; 5 Key Laboratory of Inorganic Coating Materials, Shanghai Institute of Ceramics, Chinese Academy of Sciences, Shanghai, 200050 China

**Keywords:** Reactive oxygen species, Cu_3_SnS_4_, Photocatalytic, Photothermal, Antibacterial therapy

## Abstract

**Background:**

The rapid spread of infectious bacteria has brought great challenges to public health. It is imperative to explore effective and environment-friendly antibacterial modality to defeat antibiotic-resistant bacteria with high biosafety and broad-spectrum antibacterial property.

**Results:**

Herein, biocompatible Cu_3_SnS_4_ nanoflakes (NFs) were prepared by a facile and low-cost fabrication procedure. These Cu_3_SnS_4_ NFs could be activated by visible light, leading to visible light-mediated photocatalytic generation of a myriad of reactive oxygen species (ROS). Besides, the plasmonic Cu_3_SnS_4_ NFs exhibit strong near infrared (NIR) absorption and a high photothermal conversion efficiency of 55.7%. The ROS mediated cellular oxidative damage and the NIR mediated photothermal disruption of bacterial membranes collaboratively contributed to the advanced antibacterial therapy, which has been validated by the efficient eradication of both Gram-negative *Escherichia coli* and Gram-positive methicillin-resistant *Staphylococcus aureus* strains in vitro and in vivo. Meanwhile, the exogenous copper ions metabolism from the Cu_3_SnS_4_ NFs facilitated the endothelial cell angiogenesis and collagen deposition, thus expediting the wound healing. Importantly, the inherent localized surface plasmon resonance effect of Cu_3_SnS_4_ NFs empowered them as an active substrate for surface-enhanced Raman scattering (SERS) imaging and SERS-labeled bacteria detection.

**Conclusions:**

The low cost and biocompatibility together with the solar-driven broad-spectrum photocatalytic/photothermal antibacterial property of Cu_3_SnS_4_ NFs make them a candidate for sensitive bacteria detection and effective antibacterial treatment.

**Graphical Abstract:**

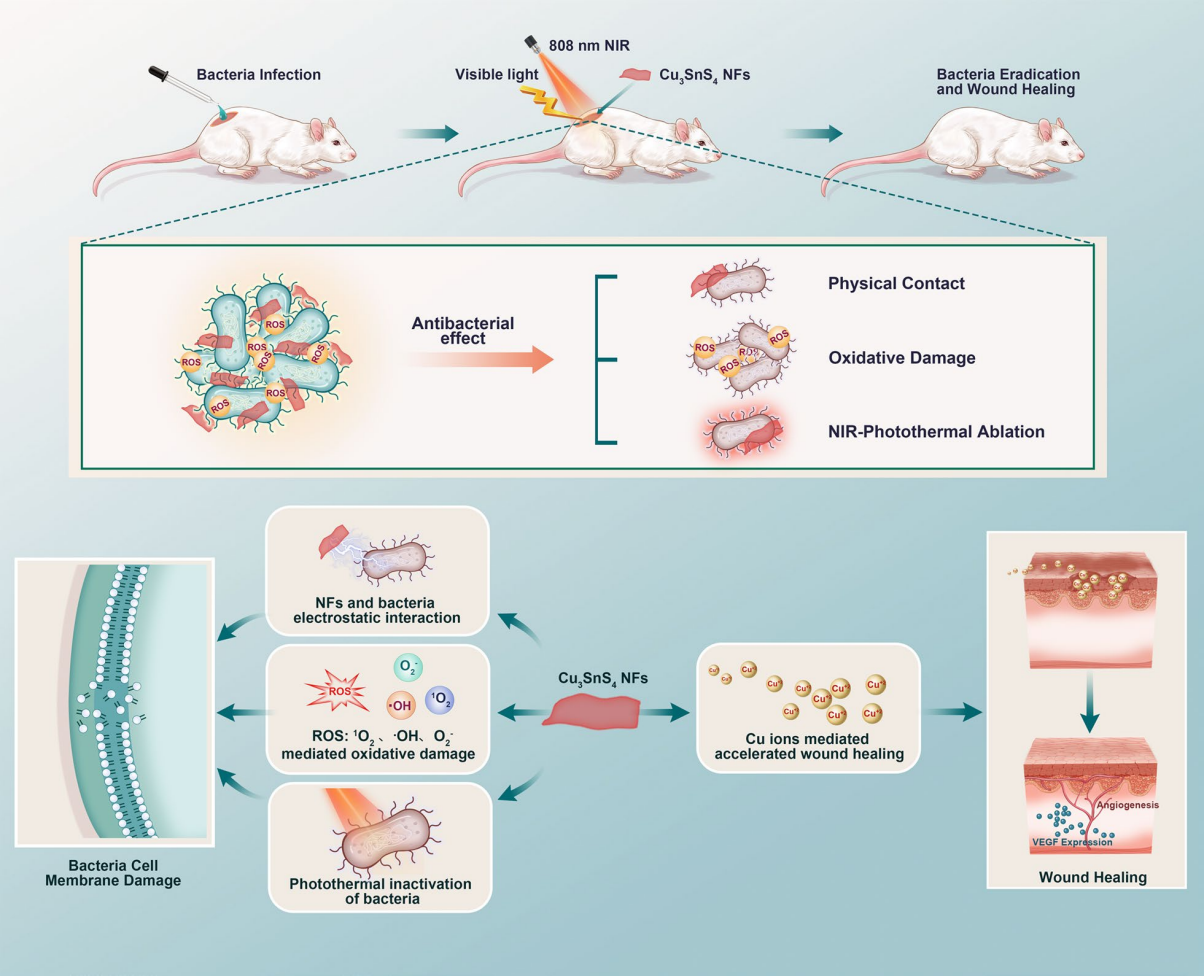

**Supplementary Information:**

The online version contains supplementary material available at 10.1186/s12951-022-01403-y.

## Background

The continuous and rapid spread of infectious bacteria has brought great challenges to public health. This challenge has been further exacerbated by the excessive use of antibiotics, which cultivates multidrug-resistant “super-bacteria” that may go out of the control of traditional antibiotics treatment [1]. During the past few years, a series of antibacterial materials, such as peptides, polymers, quaternary ammonium salts, metal oxides, and inorganic nanoparticles [2–6], have proven as bactericidal or bacteriostatic agents due to their unique antibacterial mechanisms. Although the great potential of the abovementioned antibacterial materials as alternative antibiotics, further clinical applications are limited by their potential biotoxicity and low antibacterial efficiency. It is imperative to explore more effective and environment-friendly antibacterial modality to defeat antibiotic-resistant bacteria with high biosafety and broad-spectrum antibacterial property.

Recently, photocatalytic technology has emerged as a promising option for administrating antibiotic-resistant bacteria in water disinfection [7–10]. Photocatalysis involves the use of solar light for activating photocatalysts to split water, followed by the generation of a myriad of reactive oxygen species (ROS) such as hydrogen peroxide (H_2_O_2_), hydroxyl radicals (•OH), superoxide radicals (O_2_^•−^) and singlet oxygen (^1^O_2_) [11–15]. These ROS could react with proteins, lipids, polysaccharides as well as other constituents of bacteria, leading to oxidative damage of bacterial membrane and ultimate cell death [13, 16–18]. It is noteworthy that ROS-based photocatalysis has been revealed to play a vital role in preventing bacterial biofilms formation, showing great potential for even antibiotic-resistant bacteria [19, 20]. The ideal properties for photocatalysts in antibacterial therapy are (i) facile and low-cost fabrication procedure for future mass application; (ii) environment-friendly and high biosafety to normal tissues; (iii) efficient photocatalytic reactivity with extended absorption region to achieve full spectrum solar-driven photocatalysis; (iv) broad-spectrum antibacterial property for even multidrug-resistant bacteria. Ternary Cu_3_SnS_4_ is a p-type semiconductor with a high absorption coefficient (~ 10^5^ cm^−1^) and a tunable optical band gap (0.8–1.7 eV) [21]. The narrow band gap of Cu_3_SnS_4_ enables it an extended absorption in visible and near infrared (NIR) regions. Since semiconductor photocatalysts such as ZnO, TiO_2_ and Ag-C_3_N_4_ composite [22] commonly are only active in the ultraviolet (UV) light spectrum, which occupies only 4% of the solar spectrum [7]. The higher and extended optional absorption of Cu_3_SnS_4_ in the visible region (occupies 44% of the solar spectrum) thus makes it a desired photocatalyst for visible (Vis) light activatable antibacterial applications. More importantly, the plasmonic Cu_3_SnS_4_ exhibits strong NIR absorption and high quantum efficiency, which could be converted to thermal energy and induce NIR based photothermal sterilization [23–26]. Notably, unlike the narrow bandgap metal sulfides such as CdS, PbS, ZnInS_4_, AgGaS_2_ and ZnCdS, which suffer from drawbacks of elemental toxicity (Cd and Pb) and higher costs (In and Ga). The Cu_3_SnS_4_ is consisted of low-cost and non-toxic elements of Cu, Sn and S, which have been reported to play a vital role in regulating the metabolic activity in skin pigmentation, tissue repairs, and acting as building blocks of proteins and tissues [27, 28].

**Scheme 1:**
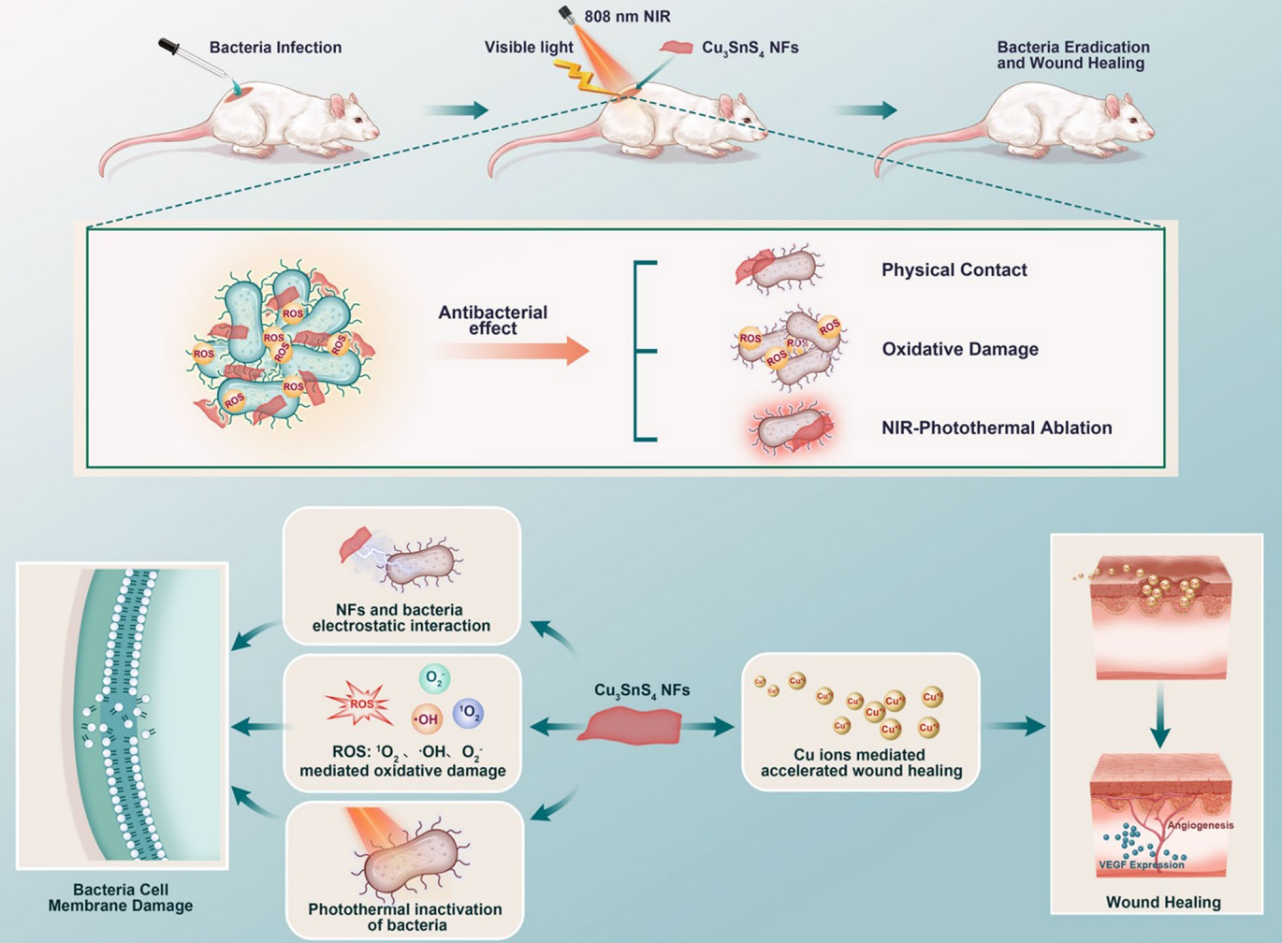
Illustration of the advanced photocatalytic and photothermal antibacterial therapy by the photo-activated Cu_3_SnS_4_ NFs. The electrostatic interaction, the ROS mediated oxidative damage, and the photothermal inactivation play a synergistic role in Cu_3_SnS_4_ NFs mediated antibacterial effect, which leads to obvious cell membrane damage to both Gram-negative *E. coli* and Gram-positive MRSA strains. Additionally, the copper ions released from the Cu_3_SnS_4_ NFs facilitate the vascular endothelial growth factor (VEGF) expression, which contributes to the revascularization of wounds and acceleration of wound healing

Herein, we presented a facile and low-cost fabrication procedure for Cu_3_SnS_4_ nanoflakes (NFs) and proposed a Vis-mediated photocatalytic and NIR-activated photothermal synergistic therapeutic strategy for combating the infections of both Gram-negative *Escherichia coli* (*E. coli*) and Gram-positive methicillin-resistant *Staphylococcus aureus* (MRSA) strains (Scheme 1). The underlying antibacterial mechanism was carefully investigated and it was proposed that the ROS mediated cellular oxidative damage and NIR mediated photothermal disruption of bacterial membranes as well as physical contact collaboratively led to the advanced antibacterial therapy. A mouse skin defect and epidermal infected model were established in vivo, showing effective bacteria eradication and favorable healing effects with negligible biotoxicity. Moreover, the inherent localized surface plasmon resonance (LSPR) effect of Cu_3_SnS_4_ NFs empowered them as an active substrate for surface-enhanced Raman scattering (SERS) imaging and SERS-labeled bacteria detection. Overall, the low cost and biocompatibility together with the solar-driven broad-spectrum photocatalytic/photothermal antibacterial property of Cu_3_SnS_4_ NFs make them a candidate for sensitive bacteria detection and effective antibacterial treatment, showing great potential for future clinic applications.

## Results and discussion

### Construction and characterization of Cu_3_SnS_4_ NFs

**Fig. 1:**
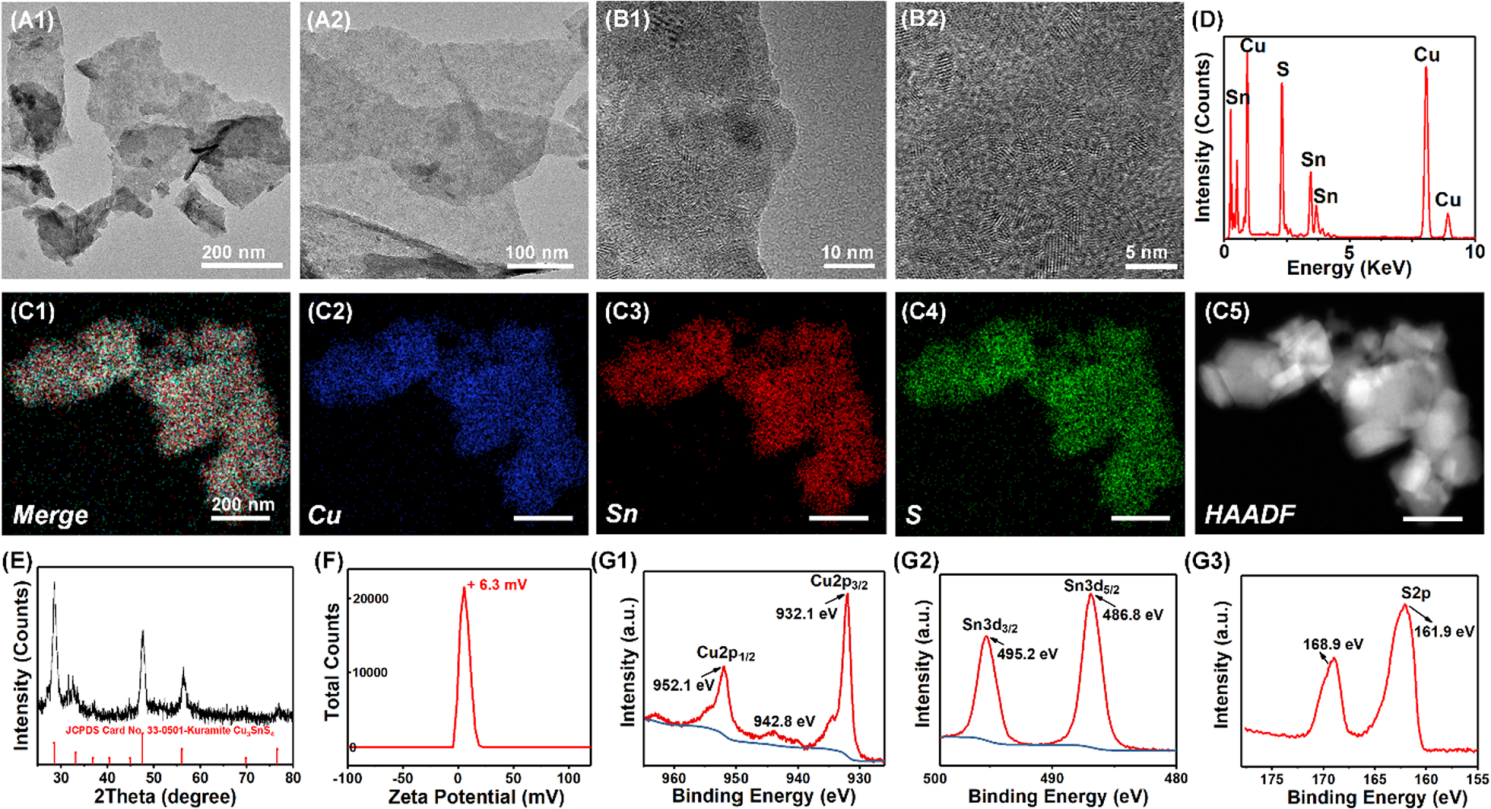
Characterization of as-prepared Cu_3_SnS_4_ NFs. **A**, **B** TEM images of Cu_3_SnS_4_ NFs. **C** Element mappings of Cu, Sn and S elements in Cu_3_SnS_4_. **D** EDS of the Cu_3_SnS_4_ NFs. **E** XRD pattern of the as-prepared Cu_3_SnS_4_ and the standard JCPDS card No. 33–0501. **F** Zeta potential of the Cu_3_SnS_4_. **G** XPS analyses of the Cu_3_SnS_4_

**Fig. 2:**
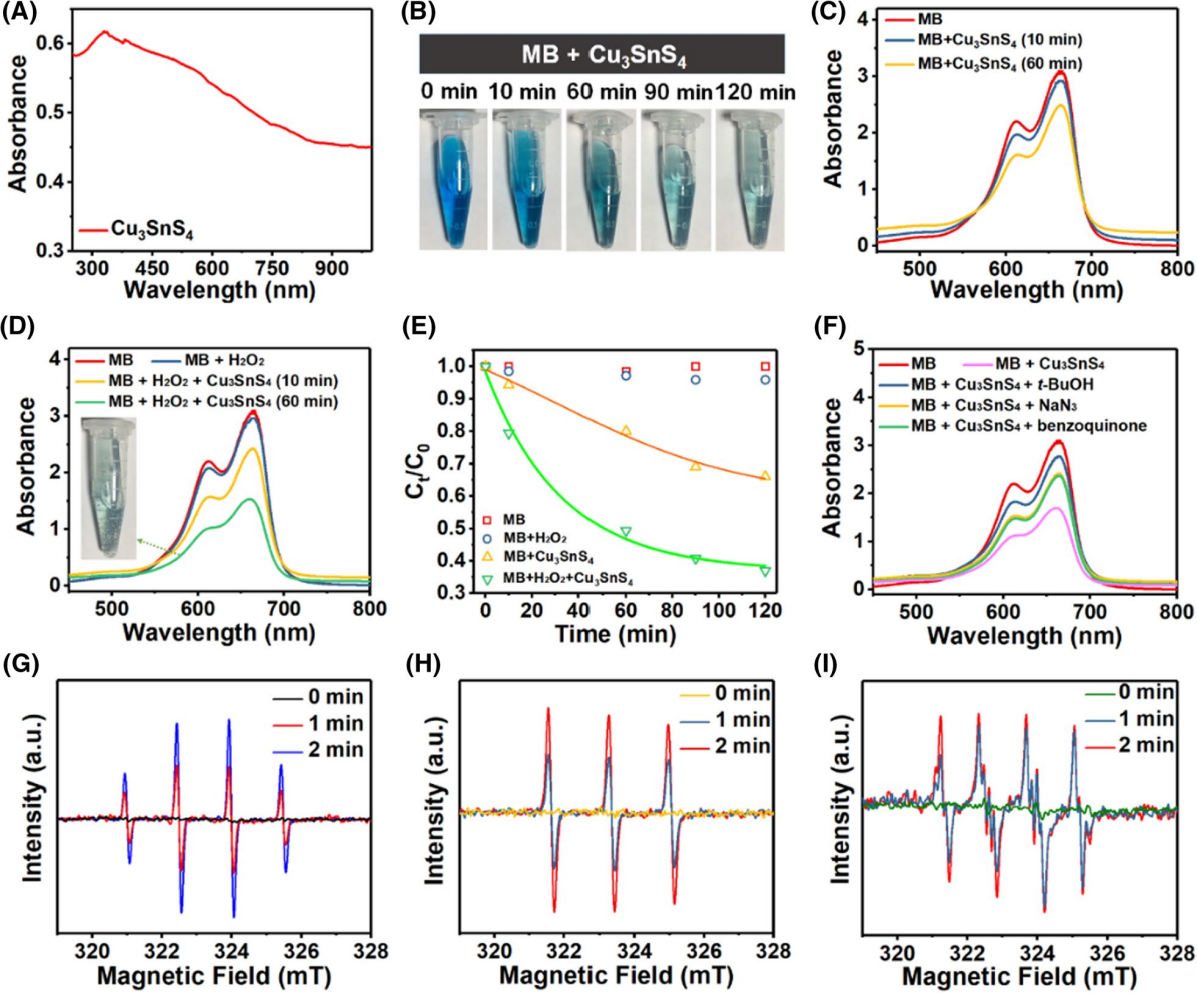
**A** The UV/Vis/NIR absorption spectrum of the Cu_3_SnS_4_ NFs. **B** Photographs of the Cu_3_SnS_4_ mediated and time-dependent degradation of MB. **C** Time-dependent UV/Vis absorption spectra of MB solution after incubation with Cu_3_SnS_4_ under visible light irradiation. **D** Time-dependent UV/Vis absorption spectra of MB solution after incubation with Cu_3_SnS_4_ and/or H_2_O_2_ under visible light irradiation. **E** Time-dependent concentration change of MB after incubation with Cu_3_SnS_4_ and/or H_2_O_2_. **F** UV/Vis absorption spectra of MB solution after incubation with Cu_3_SnS_4_ in the presence of *t*-BuOH, NaN_3_, or benzoquinone, demonstrating the influence of *t*-BuOH, NaN_3_, and benzoquinone on the Cu_3_SnS_4_-activated MB degradation. **G**–**I** EPR spectra of **G** •OH, **H** ^1^O_2_, and **I** O_2_^•−^ upon dispersion of Cu_3_SnS_4_ into deionized water under visible light irradiation for different times

**Fig. 3:**
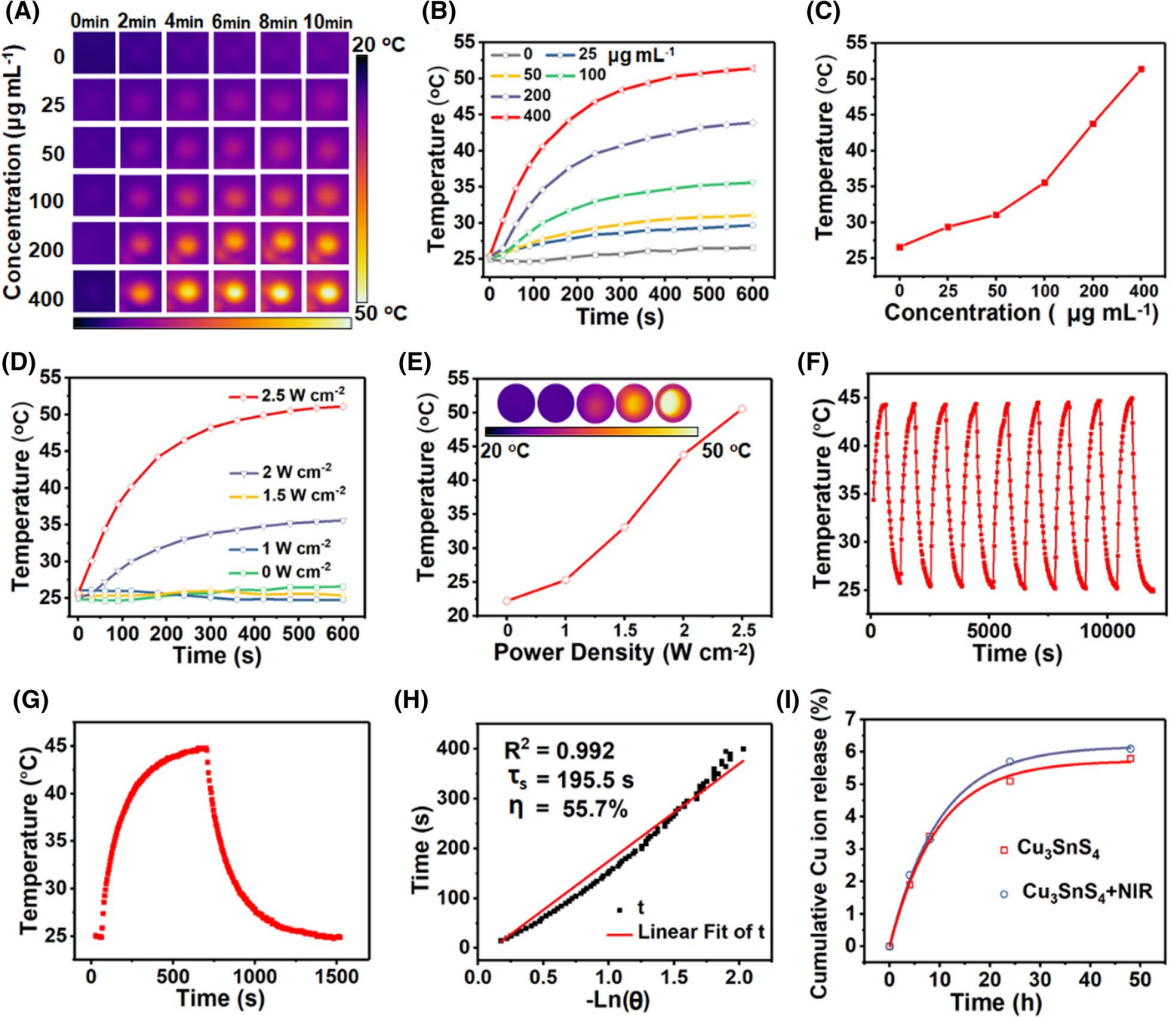
**A**–**C** Infrared thermal images and corresponding photothermal heating curves of pure water and aqueous dispersions of Cu_3_SnS_4_ at different Cu_3_SnS_4_ concentrations under continuous irradiation by a 808 nm laser with a power density of 2.5 W cm^−2^. **D**, **E** Photothermal heating curves of aqueous dispersions of Cu_3_SnS_4_ by a 808 nm laser with varied power density. **F** Temperature changes of Cu_3_SnS_4_ aqueous dispersions over nine cycles of irradiation/cooling processes. **G** The temperature profile of Cu_3_SnS_4_ aqueous dispersions irradiated with a 808-nm laser, followed by natural cooling after the laser was turned off. **H** Determination of the system time constant using linear regression of the cooling profile shown in (**G**). **I** Cu ions release from the Cu_3_SnS_4_ in PBS (pH 7.4) in the presence or absence of the NIR irradiation

Cu_3_SnS_4_ NFs were fabricated via a modified solvothermal procedure (Additional file 1: Scheme S1) [29, 30]. As shown in the transmission electron microscopy (TEM) images in Fig. 1A, nearly monodispersed foliate Cu_3_SnS_4_ NFs were produced. Notably, these NFs were built up by nanocrystals, as can be seen from the high-resolution TEM images and the selected area electron diffraction (SAED) pattern (Fig. 1B, Additional file 1: Fig. S1 and S2). The element mappings in Fig. 1C indicate that each element of Cu, Sn and S was presented and distributed in the framework of Cu_3_SnS_4_ NFs uniformly. The energy-disperse X-ray spectrum (EDS) shows the distribution of Cu, Sn and S elements without the presence of any other impurities (Fig. 1D). The atomic ratio of Cu:Sn:S is about 2.4:1:3.26, indicating Cu and S vacancies compared with the stoichiometric ratio of Cu_3_SnS_4_. The crystal structure of as-prepared Cu_3_SnS_4_ NFs was further characterized by X-ray diffraction (XRD). As shown in Fig. 1E, the diffraction peaks at 2θ values of 28.5, 47.5, and 56.0 were assigned to the (112), (220) and (132) crystal planes of tetragonal Cu_3_SnS_4_ (JCPDS card No. 33–0501). No impurities can be found in the XRD pattern, indicating the pure phase of Cu_3_SnS_4_ without secondary phases. The zeta potential analysis shows that the surface of Cu_3_SnS_4_ NFs was positively charged (+ 6.3 mV) in deionized water (Fig. 1F). X-ray photoelectron spectroscopy (XPS) was used to further explore the oxidation state of Cu, Sn and S elements in the Cu_3_SnS_4_. The XPS survey spectrum reveals the presence of Cu, Sn and S peaks (Additional file 1: Fig. S3). The binding energies of the Cu2p_3/2_ and Cu2p_1/2_ centered at 932.1 eV and 952.1 eV respectively (Fig. 1G-1), which could be typically assigned to Cu^+^ species [31]. Additionally, the Cu2p_3/2_ satellite peak at 942.8 eV could be identified as Cu^2+^ species [31], suggesting the presence of both Cu^+^ and Cu^2+^ species in the Cu_3_SnS_4_ NFs. The binding energies of the Sn3d_5/2_ and Sn3d_3/2_ centered at 486.8 eV and 495.2 eV respectively (Fig. 1G-2), corresponding to the reported values for Sn^4+^ [32]. No evidence of Sn^2+^ (binding energy at 485.2 eV) was detected in the sample. The S2p core-level spectrum indicated the binding energy at 161.9 eV (Fig. 1G-3). Additional peak at 168.9 eV was also detected, which may be attributed to the oxidized parts of the product.

### The photocatalysis mediated ROS generation from Cu_3_SnS_4_ NFs

As shown in Fig. 2A, the typical optical absorption range of Cu_3_SnS_4_ NFs crosses from the UV to NIR region (250–1000 nm), highly desirable for full spectrum solar-driven photocatalysis and photothermal antibacterial treatment [33]. The visible light mediated photocatalytic ROS generation was initially evaluated by methylene blue (MB) degradation kinetics, which was monitored by an UV/Vis spectrophotometer. Under visible light irradiation, time-dependent decolorization of MB was observed after incubation with Cu_3_SnS_4_ NFs for different times (Fig. 2B). The degradation of MB was confirmed by its characteristic absorbance at 664 nm, which decreased with the reaction proceeding (Fig. 2C). Since both Cu^+^ and Cu^2+^ are presented in the Cu_3_SnS_4_ NFs, it was expected that the Cu ions mediated Fenton-like reaction may boost the ROS generation and further exacerbate the degradation of MB [34]. As observed, the adding of tiny H_2_O_2_ (1 mM) into the reaction mixture greatly improved the time-dependent degradation efficiency (Fig. 2D, E). It was noted that almost no degradation of MB occurred in 1 mM H_2_O_2_ solution without the Cu_3_SnS_4_ catalyst, demonstrating the photocatalysis mediated generation of ROS and degradation of MB. In advanced oxidation technologies, hydroxyl radicals (·OH) are known as one of the key active species for MB degradation with a redox potential of + 2.8 V (versus NHE) in acidic media and + 1.5 V (versus NHE) in basic media [35]. To test whether ·OH was generated during the Cu_3_SnS_4_ mediated photocatalytic process, *t*-butanol (*t*-BuOH), a powerful ·OH scavenger [36], was pre-added to the reaction mixture to investigate whether the degradation could be retarded. It was found that the adding of *t*-BuOH resulted in a concentration-dependent decrease of the MB degradation but not completely quench the reaction (Fig. 2F and Additional file 1: Fig. S4). It is reasonable to speculate that other radical species may be involved in the MB degradation. Here, sodium azide (NaN_3_, 5 mM), a well-known scavenger for the singlet oxygen (^1^O_2_) [37, 38], was pre-added to the reaction mixture. As expected, the MB degradation was significantly restrained in the presence of NaN_3_, suggesting the involvement of ^1^O_2_ in the Cu_3_SnS_4_ mediated MB degradation (Fig. 2F). Similarly, the adding of benzoquinone (1 mM), a scavenger of the superoxide radical (O_2_^·−^) [39], also greatly alleviated the MB degradation, indicating the occurrence of O_2_^·−^ in the degradation. These data revealed that ·OH, ^1^O_2_, and O_2_^·−^ possibly occurred and participated in the MB degradation process. In addition, to directly prove the Cu_3_SnS_4_ mediated ROS generation, the content of ·OH, ^1^O_2_, and O_2_^·−^ was detected by electron paramagnetic resonance (EPR) using DMPO (5,5-dimethyl-1-pyrroline *N*-oxide) and TEMPO (2,2,6,6-tetramethylpiperidine-1-oxyl) as spin probes [40]. As demonstrated in Fig. 2G–I, the main active species including •OH, ^1^O_2_, and O_2_^·−^ could be produced by the Cu_3_SnS_4_ NFs under visible light irradiation. Importantly, the amount of ·OH, ^1^O_2_, and O_2_^·−^ all gradually increased with the prolonged irradiation time. These ROS may further attack bacteria around the photocatalyst and contribute to efficient antibacterial treatment.

### The NIR mediated hyperthermia of Cu_3_SnS_4_ NFs

Cu_3_SnS_4_ possesses obvious localized surface plasmon resonances (LSPR) in the NIR band [29, 41]. It was speculated that the strong NIR absorption of Cu_3_SnS_4_ NFs observed in this work could be attributed to LSPR, which may further lead to photothermal effect for antibacterial treatment. Here, different concentrations of Cu_3_SnS_4_ NFs were dispersed in aqueous solutions and exposed to an 808 nm NIR laser irradiation for different times. Figure 3A–C clearly displays the concentration and irradiation time-dependent photothermal conversion of Cu_3_SnS_4_ NFs. The solution temperature was precisely controlled between 25 °C and 52 °C by varying the Cu_3_SnS_4_ concentration or the irradiation time of the NIR light. In contrast, the temperature change of pure water was less than 2 °C under otherwise identical experimental conditions. Importantly, the photothermal conversion of Cu_3_SnS_4_ NFs was also found to be laser power density-dependent, showing distinct temperature increase when promoting the laser power density from 0 W cm^−2^ to 2.5 W cm^−2^ (Fig. 3D, E). Nine successive cycles of heating/cooling processes demonstrated the stable photothermal conversion capability, which is of key importance for photothermal agents during photothermal treatment (Fig. 3F). The photothermal conversion efficiency of Cu_3_SnS_4_ NFs was calculated to be 55.7% (Fig. 3G, H). The Cu ions released from the Cu_3_SnS_4_ NFs were evaluated by inductively coupled plasma optical emission spectrometry (ICP-OES). 5.1% of Cu ions was released from the Cu_3_SnS_4_ NFs within 24 h and 5.8% within 48 h in PBS (pH 7.4), respectively (Fig. 3I). Besides, it was found that the NIR irradiation (808 nm, 2.0 W cm^−2^, 10 min) did not show distinct interference on the release of Cu ions.

### In vitro antibacterial activity

**Fig. 4:**
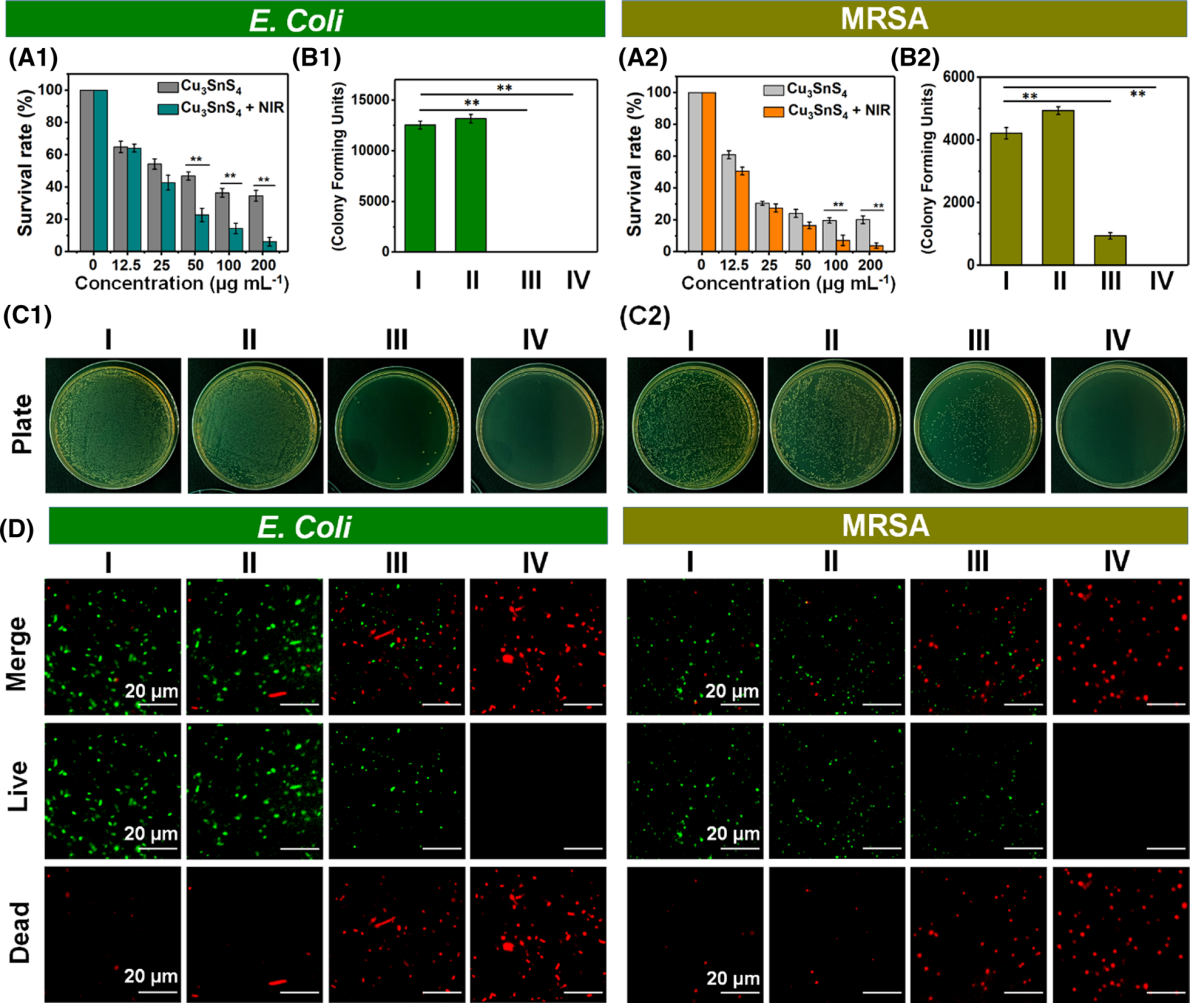
**A** Growth survival of (A1) *E. coli* and (A2) MRSA bacteria. Data are presented as means ± SDs (n = 6). ^*^p < 0.05, ^**^p < 0.01. **B** CFU amount of (B1) *E. coli* and (B2) MRSA bacteria. **C** Photographs of (C1) *E. coli* and (C2) MRSA bacterial colonies formed on lysogeny broth (LB)-agar plates. **D** Representative CLSM images of live/dead staining assay for *E. coli* (left) and MRSA (right) after different treatments via SYTO9 (green, viable bacteria) and PI (red, dead bacteria) staining. Group I: control group in the absence of NFs and NIR irradiation; Group II: NIR group with the irradiation of NIR laser (808 nm, 1.5 W cm^−2^, 10 min) in the absence of NFs; Group III: Cu_3_SnS_4_ group with the treatment with Cu_3_SnS_4_ NFs under no laser irradiation; Group IV: Cu_3_SnS_4_ + NIR group with the treatment with Cu_3_SnS_4_ NFs plus NIR irradiation (808 nm, 1.5 W cm^−2^, 10 min). All groups were exposed to visible light for 20 min

Considering the excellent properties of Cu_3_SnS_4_ NFs as described above, we set out to examine the possibility of the NFs as a potential photocatalytic agent in combination with the photothermal performance for synergistic elimination of bacteria. Here, the synergistic antibacterial activities of Cu_3_SnS_4_ NFs against both Gram-negative and Gram-positive bacteria were evaluated using *E. coli* and MRSA as model strains, respectively. The relative bacterial cells survival was calculated and the colony forming unit (CFU) plate counting method was used to assess the photo-activated disinfection ability of Cu_3_SnS_4_ NFs (Additional file 1: Fig. S5). As shown in Fig. 4A and Additional file 1: Fig. S6, both bacteria of *E. coli* and MRSA did not show apparent response to only visible light treatment in the absence of the Cu_3_SnS_4_ NFs and the cell survival was accordingly used as the control group (Group I). In contrast, the bacterial cells survival dramatically decreased to 46.8% (*E. coli*) and 24.1% (MRSA) after treating with Cu_3_SnS_4_ at the concentration of 50 μg mL^−1^ under visible light for 20 min (Group III), demonstrating the Cu_3_SnS_4_ NFs mediated inactivation of both bacteria strains. Notably, the bacterial cells survival further decreased to 22.8% (*E. coli*) and 16.5% (MRSA) after the Cu_3_SnS_4_ plus 808 nm NIR treatment (10 min) under otherwise identical conditions (Group IV). Since NIR alone (Group II, 808 nm, 1.5 W cm^−2^, 10 min) did not show any antibacterial effect against both strains (Additional file 1: Fig. S7), the enhanced antibacterial effect after the Cu_3_SnS_4_ plus NIR treatment thus could be attributed to the NIR activated and synergistic photothermal effect of Cu_3_SnS_4_. Moreover, the photo-activated disinfection activity of Cu_3_SnS_4_ NFs could be further enhanced by increasing the dose of the NFs (Fig. 4A). The bacterial cells survival further decreased to 36.4% for *E. coli* and 19.6% for MRSA when promoting the Cu_3_SnS_4_ NFs dose to 100 μg mL^−1^. The CFU plate counting provides a more intuitive characterization of the disinfection ability of Cu_3_SnS_4_ NFs (Fig. 4B, C). The antibacterial results under only NIR laser irradiation (group II) was almost the same as that in the control group (group I). In contrast, with the Cu_3_SnS_4_ NFs treatment, a drastic decrease of MRSA colonies was observed and even no *E. coli* colonies could be detected under only visible light irradiation for 20 min (group III). More significant disinfection efficiency and complete inhibition of bacterial growth were detected in both bacterial models after the Cu_3_SnS_4_ NFs plus NIR treatment (group IV), demonstrating the remarkable antibacterial ability of photo-activated Cu_3_SnS_4_ NFs (Fig. 4B, C). The tiny Cu ions released from the Cu_3_SnS_4_ NFs were found to play a negligible role on the antibacterial activity of the NFs, as verified by the extract liquid results (Additional file 1: Fig. S8). Fluorescence staining of live/dead cells was performed to further verify the feasibility of eliminating bacteria with different treatments. As shown in the confocal laser scanning microscopy (CLSM) images in Fig. 4D, compared with the green fluorescence displayed in both the control group (group I) and the NIR group (group II), a number of dead cells (red fluorescence) were clearly detected in both strains after incubation with Cu_3_SnS_4_ NFs even without laser irradiation (group III), which was consistent with the bacterial cell survival results. As expected, treatment with Cu_3_SnS_4_ NFs plus NIR irradiation induced almost complete death of both bacterial cells, indicating their synergistic and stronger cell-killing effect (group IV).

**Fig. 5:**
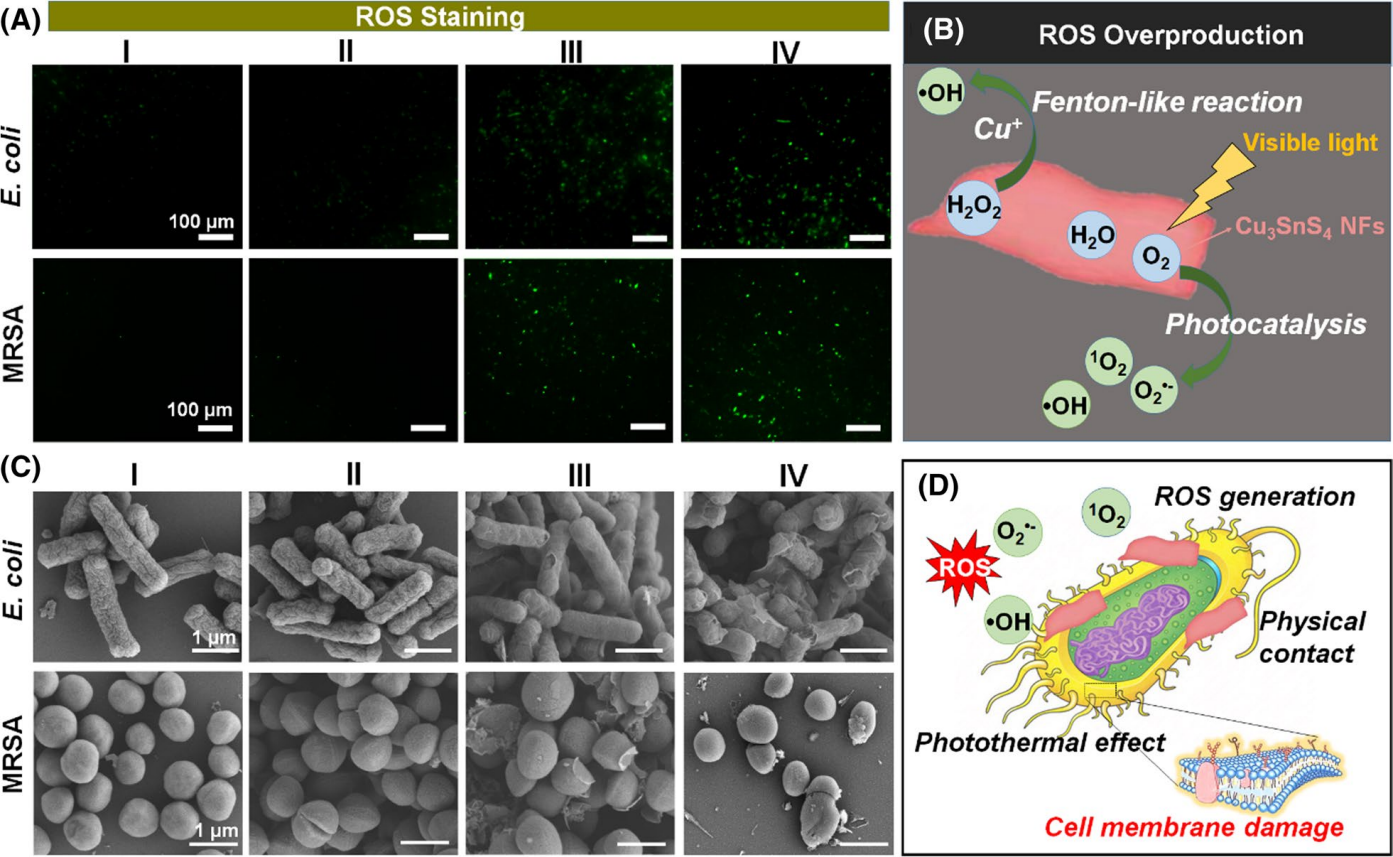
**A** Intracellular ROS level indicated by DCFH-DA fluorescence staining after diverse treatments. **B** Scheme of the ROS overproduction induced by the Cu ions mediated Fenton-like reaction and the visible light mediated photocatalytic process. **C** SEM micrographs of *E. coli* and MRSA after different treatments. **D** Scheme of the interaction of the physical contact, ROS generation and photothermal effects, which collaboratively led to the damage of bacteria cell membrane. Group I: control group in the absence of NFs; Group II: NIR group with the irradiation of NIR laser (808 nm, 1.5 W cm^−2^, 10 min) in the absence of NFs; Group III: Cu_3_SnS_4_ group with the treatment with Cu_3_SnS_4_ NFs under no laser irradiation; Group IV: Cu_3_SnS_4_ + NIR group with the treatment with Cu_3_SnS_4_ NFs plus NIR irradiation (808 nm, 1.5 W cm^−2^, 10 min). All groups were exposed to visible light for 20 min

In this study, we rationalized the efficient antimicrobial activity of Cu_3_SnS_4_ NFs on the basis of the photocatalysis-mediated ROS generation, which further induced the oxidative stress to kill bacterial cells. To highlight this property, the ROS level in MRSA bacterial cells were explored by 2′,7′-dichlorodihydrofluorescein diacetate (DCFH-DA) staining, which acts as a commonly used method to evaluate the overall oxidative stress level in cells. As exhibited in Fig. 5A and Additional file 1: Fig. S9, bacteria treated with Cu_3_SnS_4_ NFs with or without NIR irradiation displayed an elevated level of intracellular ROS, which may contribute to the observed antibacterial efficacy of Cu_3_SnS_4_ NFs. Since both Cu^+^ and Cu^2+^ are presented in the Cu_3_SnS_4_ NFs, it was expected that the Cu ions mediated Fenton-like reaction together with the visible light mediated photocatalysis may collaboratively result in the ROS overproduction (Fig. 5B). To further investigate the antibacterial mechanism of photo-activated Cu_3_SnS_4_ NFs, the cell morphologies of both *E. coli* and MRSA following different treatments were studied by scanning electron microscopy (SEM, Fig. 5C). Indeed, in contrast to the well-maintained cell morphology in the control group (group I) and NIR group (group II), distorted cell morphology with lesions and holes on the bacterial cell wall/membranes were observed in both bacterial strains after treatment with Cu_3_SnS_4_ NFs with or without NIR irradiation (group III and group IV). The photogenerated ROS and hyperthermia were presumed to contribute to the cell membrane damage jointly. Besides, a mass of NFs were discovered on the surface of bacteria. Since the surface of bacteria are commonly negatively charged due to the presence of teichoic acids in the cell wall of Gram-positive bacteria and lipopolysaccharide in the outer membrane of Gram-negative bacteria [19], it could be speculated that the electrostatic interaction between the positively charged NFs and the negatively charged bacteria would facilitate the preferential absorption (physical contact) of NFs and reinforce the photocatalysis-based antibacterial efficacy by compromising bacterial membrane integrity [42] (Fig. 5D).

### In vivo antibacterial activity

**Fig. 6:**
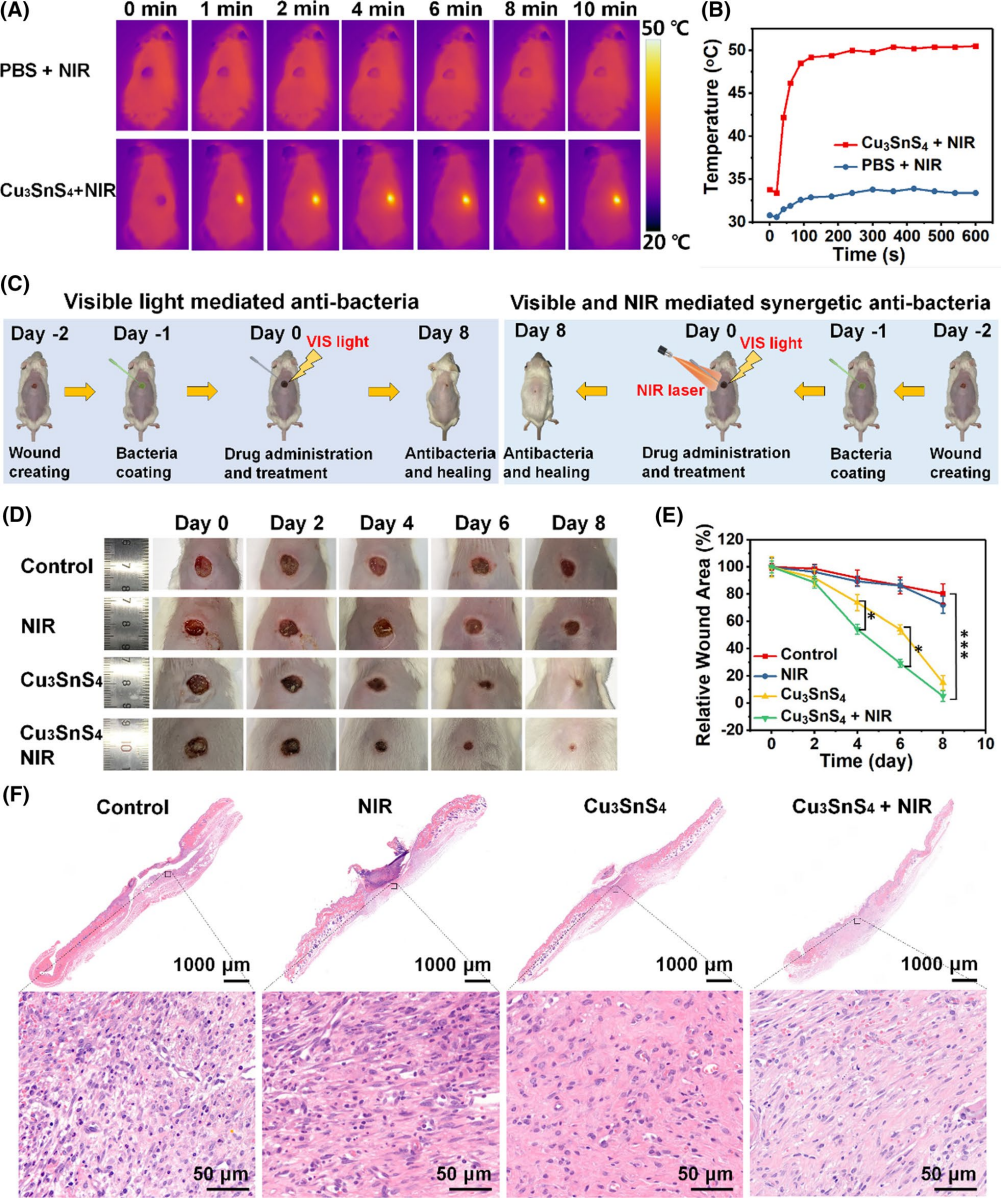
**A** In vivo thermal infrared images of mice after different treatments. Mice were treated with PBS or Cu_3_SnS_4_ NFs followed by NIR laser irradiation for different times. **B** Corresponding temperature change of wounds of mice after treatment with PBS or Cu_3_SnS_4_ NFs followed by 808 nm laser irradiation. **C** Scheme of the photo-activated antibacterial treatment processes. **D** Representative photographic images of ICR mice with MRSA-infected wounds after different treatments for 8 days: control (treated with PBS), NIR (treated with PBS followed by NIR irradiation), Cu_3_SnS_4_ (treated with Cu_3_SnS_4_ NFs), and Cu_3_SnS_4_ + NIR (treated with Cu_3_SnS_4_ NFs followed by NIR irradiation). **E** Corresponding changes of wound size in each group during the 8 days’ treatments. Data are presented as means ± SDs (n = 6). ^*^p < 0.05, ^***^p < 0.001. **F** H&E staining of the dermal wound tissues on day 8 after different treatments. For NIR irradiation, 808 nm laser (1.0 W cm^−2^, 10 min) were used

The in vitro biocompatibility of as-prepared Cu_3_SnS_4_ NFs was evaluated prior to their in vivo biomedical applications. The cytotoxicity of Cu_3_SnS_4_ NFs to human umbilical vein endothelial cells (HUVEC) and mouse skin fibroblast L929 cells were evaluated, showing no significant toxicity in the wide concentration range of 10–100 μg mL^−1^ within 24 h and 48 h (Additional file 1: Fig. S10). To assess the in vivo antibacterial efficacy of Cu_3_SnS_4_ NFs, ICR mice with a MRSA-infected cutaneous wound model were fabricated. Initially, the in vivo photothermal effect was explored and shown in Fig. 6A. In contrast to the control group, which showed no distinct change of temperature in the wound area of mice (33.8 °C after 10 min of laser treatment), significantly increased temperature was found in the wound area of the mice treated with Cu_3_SnS_4_ NFs under the NIR laser irradiation. To be noted, the temperature in the wound area was irradiation time-dependent, which could rapidly reach 49.2 °C in 2 min and maintain around 50 °C in the following 8 min (Fig. 6B). Despite the hyperthermia, no obvious normal tissue damage was observed due to the specific and focused laser irradiation toward the wound area. Then, the photo-activated skin wound-healing ability of Cu_3_SnS_4_ NFs in wound-infecting mice was evaluated. The treatment process is shown in Fig. 6C. A rounded cutaneous wound model (6 mm in diameter) was constructed in male ICR mice. Then, MRSA, a common cause of skin infections, was used to construct the wound infecting model. Afterwards, the MRSA*-*infected mice were divided into four groups (n = 6 for each group) and treated with (i) PBS (control group), (ii) PBS followed by 808 nm NIR laser irradiation (NIR group), (iii) Cu_3_SnS_4_ dispersed in PBS (Cu_3_SnS_4_ group), (iv) Cu_3_SnS_4_ dispersed in PBS followed by 808 nm NIR laser irradiation (Cu_3_SnS_4_ + NIR group), respectively. Mice in all groups were exposed to visible light during the treatment. The size of the wound and weight of each mouse were recorded every two days. As shown in Fig. 6D, mice treated with PBS or NIR alone did not show distinct healing of wound despite the presence of scars in both groups (control and NIR group). Significantly, the wound area of mice treated with Cu_3_SnS_4_ (Cu_3_SnS_4_ group) was generally smaller, showing around 93.5% recovery on day 8 (Fig. 6E). More importantly, mice treated with Cu_3_SnS_4_ plus NIR irradiation (Cu_3_SnS_4_ + NIR group) displayed more pronounced recovery of wound. The trauma area on day 4 has exhibited significant difference as compared to the Cu_3_SnS_4_ group (^*^p < 0.05, Fig. 6E), demonstrating the promoted antibacterial and wound healing efficiency based on the collaborative photocatalytic and photothermal antibacterial effects. To further investigate the wound healing process, the wound skin in each group was harvested and exposed to Hematoxylin and eosin (H&E) staining after the treatment. As shown in Fig. 6F, the wound boundary between the wound and normal tissue could be observed clearly from the histological staining results. The keratinocytes migrated to the wound area from the normal tissue and scars became significantly smaller and even vanished after the treatment with Cu_3_SnS_4_ NFs or Cu_3_SnS_4_ NFs plus NIR irradiation, whereas incomplete dermal layer was still observed in the control and NIR groups. Besides, the histological slices of both the control group and NIR group presented a number of inflammatory cells, predominantly neutrophils and mononuclear cells adhered to the stratified squamous epithelium. In contrast, intact epidermal layer accompanied with dramatic decrease of the infiltration of inflammatory cells was found after the treatment with Cu_3_SnS_4_ or Cu_3_SnS_4_ plus NIR irradiation, further indicating the efficiency of the photo-activated antibacterial effect of the Cu_3_SnS_4_ NFs.

### In vivo evaluation of collagen deposition and angiogenesis in wounds

**Fig. 7:**
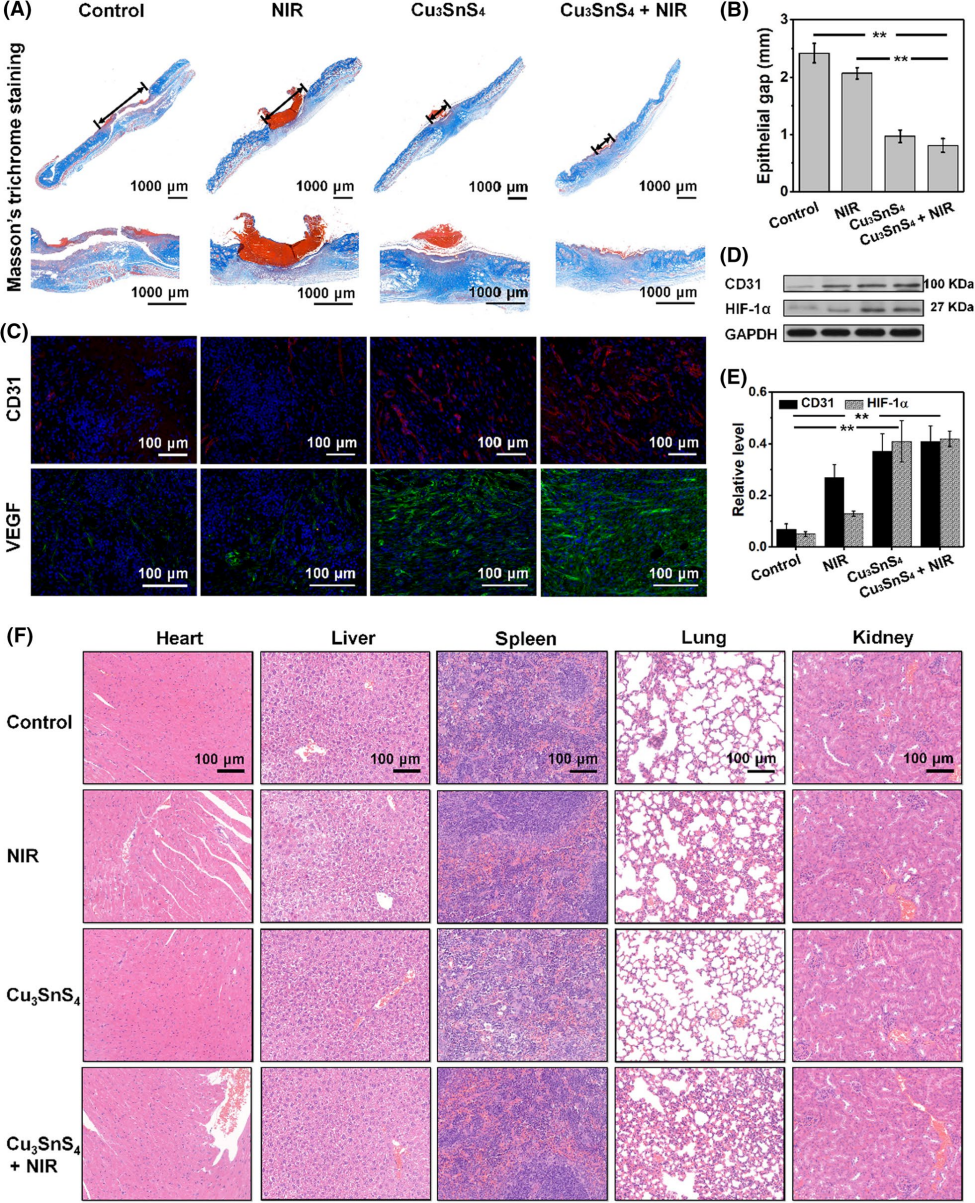
**A** Histopathological analysis of Masson’s trichrome staining of wounds in mice on day 8 after different treatments. **B** Corresponding epithelial gap in each group after the treatment. **C** Representative histological images of CD31 and VEGF immunofluorescence staining of wound tissue in each group at the end of the 8 days’ treatments. **D** Western blot analyses of CD31 and HIF-1α expressions in wound of mice in each group at the end of 8 days’ treatments. **E** Corresponding quantitative level of CD31 and HIF-1α expressions in each group. **F** Representative H&E staining images of major organs after different treatments

Wound healing is a dynamic process that involves the proliferation of fibroblasts, deposition of collagen fibers, angiogenesis, formation of granulation tissue, scar formation, wound contraction and epithelialization [43]. The synthesis and deposition of collagen is a critical factor in wound healing. Cu ions have been recognized as a cofactor to lysyl oxidase, which stimulates the expression of matrix metalloproteinase-2 and collagen in fibroblasts, facilitating wound healing [44–46]. Histological analysis of Masson's trichrome stained sections showed extensive collagen deposition and a smaller granulation tissue gap in the wounds after the treatment with Cu_3_SnS_4_ (Fig. 7A, B). Importantly, more wavy collagen fibers were observed in the wounds of the Cu_3_SnS_4_ + NIR group, demonstrating the favourable extracellular matrix remodeling of granulation tissue and regeneration of the epidermis [47]. Further, immunofluorescence staining for CD31, a transmembrane protein expressed early in vascular development, was used to evaluate the newly formed vessels [48]. It showed that the density of newly formed vessels in the wounds of the Cu_3_SnS_4_ and Cu_3_SnS_4_ + NIR group was more significant compared with the other two groups (Fig. 7C). The Western blot analysis of CD31 was in concordance with the immunofluorescence staining results, further confirming the promoted vessel number after the treatments (Fig. 7D). Moreover, it has been suggested that hypoxia plays a critical role in cell recruitment, cell differentiation and blood vessel formation [49]. Hypoxia inducible factor-1α (HIF-1α) has been identified to initiate the transcription of hypoxia sensitive genes including vascular endothelial growth factor (VEGF) under hypoxic conditions [50]. Stabilization and activation of HIF-1α expression was thus suggested as a potential strategy to promote neovascularization [51]. The HIF-1α expression and VEGF secretion in wounds after the treatment were then investigated, trying to figure out the angiogenesis during the wound healing process. The HIF-1α expression, as determined by the Western blot analysis, was promoted both in the Cu_3_SnS_4_ and Cu_3_SnS_4_ + NIR group as compared with the other two groups (Fig. 7D, E). Moreover, the immunofluorescence staining of VEGF showed more significant expression of VEGF and therefore more new blood vessel formation after the Cu_3_SnS_4_ and Cu_3_SnS_4_ + NIR treatment (Fig. 7C). The promotion of angiogenesis was expected to allow more supply of oxygen and nutrients as well as accelerated migration of requisite cells and humoral factors into wounds, which leads subsequently to the increased formation of granulation tissue, collagen synthesis and eventually improved wound healing [45].

Furthermore, the in vivo biosafety after the Cu_3_SnS_4_ and/or NIR treatment was evaluated. The body weight of mice in each group during the whole treatment process did not show distinct difference (Additional file 1: Fig. S11). Besides, the main blood biochemistry and hematology analyses of mice in each group after the treatment were conducted, suggesting normal functions in liver and kidney (Additional file 1: Fig. S12). The routine blood test was further assessed to monitor the systemic inflammatory activity, showing no significant differences as compared with the control group (Additional file 1: Fig. S13). Moreover, no visible damage, inflammation, or abnormality were observed in the histological evaluation of major organs including heart, liver, spleen, lung, and kidney (Fig. 7F). Collectively, the biosafety analyses substantiate that the photo-activated antibacterial treatment showed no obvious side-effect to mice in vivo.

### Cu_3_SnS_4_ NFs as an active SERS substrate for the detection of *E. coli *in vitro

**Fig. 8:**
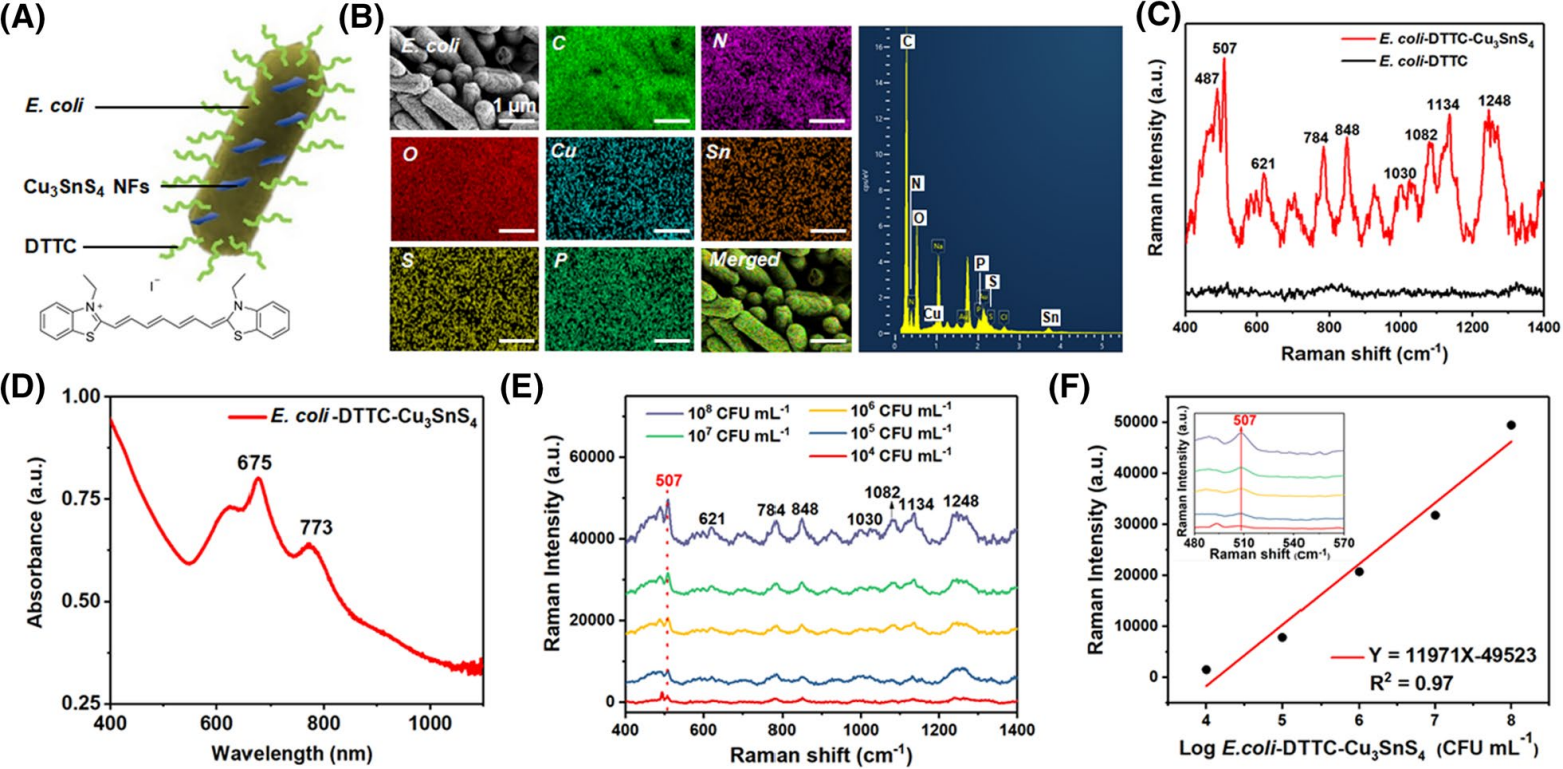
**A** Illustration of the *E. coli*-DTTC-Cu_3_SnS_4_. **B** The SEM and EDS element mapping of the *E. coli*-DTTC-Cu_3_SnS_4_. **C** The Raman spectra of *E. coli*-DTTC and *E. coli*-DTTC-Cu_3_SnS_4_. **D** The UV/Vis/NIR absorption spectrum of the *E. coli*-DTTC-Cu_3_SnS_4_. **E** In vitro SERS spectra of *E. coli*-DTTC-Cu_3_SnS_4_ under the excitation of a 785 nm laser. **F** Raman peak intensity versus Log *E. coli-*DTTC-Cu_3_SnS_4_ concentration at 507 cm^−1^

Surface-enhanced Raman scattering (SERS) imaging is a highly sensitive tool that allows direct identification of target analytes in proximity to the surface of plasmonic nanoparticles [52]. Due to its high sensitivity, chemical specific, high resolution, and non-destructive characteristics, SERS imaging has demonstrated applications in biomedicine as a promising tool for real-time bacteria detection [53]. Cu_3_SnS_4_ compound is a p-type semiconductor due to the presence of copper vacancies, which further endows Cu_3_SnS_4_ with localized surface plasmon resonance (LSPR) in the NIR region [54]. Indeed, a wide absorption band in the NIR region associated with LSPR is clearly observed in the UV/Vis/NIR spectrum of Cu_3_SnS_4_ NFs. The natural excitation of Cu_3_SnS_4_ LSPRs in the NIR paves the way for the employment of Cu_3_SnS_4_ NFs as SERS substrate for enhanced signals of Raman imaging. Here, we set out to explore the possibility of utilizing Cu_3_SnS_4_ NFs as a SERS substrate for the determination of bacteria content. *E. coli* was used as the model bacteria and tagged with Raman reporter molecules of 3,3′-diethylthiatricarbocyanine iodide (DTTC), followed by the immobilizing of Cu_3_SnS_4_ NFs (designated as *E. coli*-DTTC-Cu_3_SnS_4_). The as-prepared *E. coli*-DTTC-Cu_3_SnS_4_ is illustrated in Fig. 8A. The EDS analysis of the *E. coli*-DTTC-Cu_3_SnS_4_ showed well-merged element images of C, N, O, P, Cu, Sn, and S elements, confirming the presence of both DTTC and Cu_3_SnS_4_ on the bacteria. The *E. coli*-DTTC-Cu_3_SnS_4_ were subsequently exposed to a 785 nm laser with 10 mW laser power, 5 s acquisition time, and a 50 × object lens. Significant enhancement of characteristic Raman signals of the DTTC molecule around 507, 621, 784, 848, 1082, 1134, and 1248 cm^−1^ was observed after the coupling of Cu_3_SnS_4_ NFs [55, 56], while negligible Raman signal was captured in equivalent *E. coli*-DTTC without Cu_3_SnS_4_ nanostructure (Fig. 8C). This result clearly demonstrates the possibility of Cu_3_SnS_4_ NFs as SERS substrate for the enhancement of Raman probe signals. As can be seen from the UV/Vis/NIR spectrum of *E. coli*-DTTC-Cu_3_SnS_4_ (Fig. 8D and Additional file 1: Fig. S14), the LSPR peak of *E. coli*-DTTC-Cu_3_SnS_4_ was in proximity with the wavelength of the excitation laser for SERS imaging (785 nm). The enhancement thus could be attributed to the LSPR on the Cu_3_SnS_4_ NFs under the excitation of 785 nm laser beam, which strengthened local electromagnetic field and led to enhanced Raman probe signals [57]. We subsequently demonstrate the high sensitivity of utilizing Cu_3_SnS_4_ NFs for SERS based *E. coli* detection. A series of *E. coli* tagged with DTTC at concentrations from 10^8^ CFU mL^−1^ to 10^3^ CFU mL^−1^ were coupled with Cu_3_SnS_4_ NFs, followed by subjection to SERS detection. As shown in Fig. 8E and Additional file 1: Fig. S15, the SERS labeled bacteria with different cell counts from 10^8^ CFU mL^−1^ to 10^4^ CFU mL^−1^ could be clearly delineated with low signal-to-background ratio. Using the Raman shift of DTTC molecule at 507 cm^−1^ for detection, *E. coli* could be detected at a minimal concentration of 10^4^ CFU mL^−1^. Figure 8F displays the intensity of the SERS peak at 507 cm^−1^ as a function of logarithmic concentrations of *E. coli*-DTTC-Cu_3_SnS_4_. A linear dependence was found with concentrations of *E. coli*-DTTC-Cu_3_SnS_4_ ranging from 10^4^ CFU mL^−1^ to 10^8^ CFU mL^−1^, corresponding to doses of Cu_3_SnS_4_ ranging from 75.5 μM to 325.7 μM (Additional file 1: Fig. S16). Compared with commom noble metals (Ag and Au) nanostructure-based substrates, the fabrication procedure of Cu-based Cu_3_SnS_4_ NFs is facile and low-cost, thus representing a promising active substrate for SERS based bacteria detection.

## Conclusions

In conclusion, photo-activatable Cu_3_SnS_4_ NFs were prepared for advanced antibacterial therapy by inducing efficient bacteria eradication and accelerated wound healing. The Cu_3_SnS_4_ NFs could be activated by visible light, leading to effective overproduction of ROS in bacterial cells. Besides, the high photothermal conversion efficiency of Cu_3_SnS_4_ NFs induced NIR light (808 nm) controlled hyperthermia in bacteria. Importantly, the electrostatic interaction between the positively charged Cu_3_SnS_4_ NFs and the negatively charged bacteria facilitated the physical contact mediated bacteria killing. Consequently, the physical contact and ROS mediated cellular oxidative damage as well as the NIR mediated photothermal disruption of bacterial membranes played a synergistic role in the efficient eradication of both *E. coli* and MRSA in vitro and in vivo. On the other hand, the exogenous ions metabolism from the Cu_3_SnS_4_ NFs significantly up-regulated the expression of HIF-1α, CD31 and VEGF, leading to increased formation of granulation tissue, collagen synthesis and eventually improved wound healing. Additionally, the Cu_3_SnS_4_ NFs can be used as an active SERS imaging substrate for SERS-labeled bacteria detection in vitro. The sensitive bacteria detection and the solar-driven broad-spectrum antibacterial therapeutic effects as well as the high biocompatibility and therapeutic biosafety endow Cu_3_SnS_4_ NFs with potentiality for future clinical transformation.

## Methods

### Materials

Copper acetate hydrate (CuAc_2_·H_2_O), stannic chloride pentahydrate (SnCl_4_·5H_2_O), hydrogen peroxide (H_2_O_2_, 30 wt%), thioacetamide (TAA), and N,N-dimethylformamide (DMF) were obtained from Sinopharm Chemical Reagent Co., Ltd. Sodium azide (NaN_3_), *t*-butanol (*t*-BuOH), benzoquinone, and methylene blue (MB) were purchased from Aladdin Chemical Reagent Co., Ltd. 5,5-dimethyl-1-pyrroline N-oxide (DMPO) and 2,2,6,6-tetramethylpiperidine-1-oxyl (TEMPO) were bought from Adamas Reagent, Ltd. All reagents were purchased as received and used without any further purification.

### Preparation and characterization of Cu_3_SnS_4_ NFs

The Cu_3_SnS_4_ NFs were prepared by a facile solvothermal strategy. In a typical synthesis, 0.06 mmol CuAc_2_·H_2_O, 0.04 mmol SnCl_4_·5H_2_O and 0.08 mmol thioacetamide (TAA) were mixed in 10 ml N,N-dimethylformamide (DMF). The mixture was purged with argon and stirred for 2 h under 140 °C. Then, the reaction mixture was transferred to a stainless-steel autoclave and the reaction was processed at 220 °C for 12 h. After completing the reaction, the mixture was naturally cooled to room temperature and centrifuged at 5000 rpm for 10 min. The precipitate was washed with acetone and deionized water to remove by-products. The obtained black powder was dried at 80 °C for 12 h and used for further characterization.

### Characterization

Transmission electron microscopy (TEM), high resolution-TEM (HRTEM), energy dispersive X-ray spectroscopy (EDS), and the corresponding element mapping analyses were conducted on a JEM-2100F electron microscope. Zeta potential was determined on Malvern Zetasizer Nanoseries (Nano ZS90). X-ray photoelectron spectroscopy (XPS) was acquired on an Axis Ultra DLD spectrometer (Kratos, UK). UV–visible (UV/Vis) absorbance spectra were obtained on a UV-3101 Shimadzu spectroscope. The quantitative elemental analysis was carried out on an inductively coupled plasma optical emission spectrometer (ICP-OES, Agilent 700 Series, USA). Powder X-ray diffraction (XRD) pattern was tested on a Rigaku Ultima IV diffractometer. The electron paramagnetic resonance (EPR) was conducted on a Bruker EMXplus X-band EPR.

### The photocatalysis mediated ROS generation from Cu_3_SnS_4_ NFs

The photocatalysis mediated ROS generation was evaluated using the methylene blue (MB) probe. Briefly, 4 mL of methylene blue (MB, 10 μg mL^−1^) aqueous solution was added to 6 mL of the aqueous solution containing 0.6 mg of Cu_3_SnS_4_ NFs and shaken slightly under light. 0.5 mL of the reaction suspension was then taken out, diluted with water, and detected by a UV/Vis spectrometer at 664 nm periodically. The MB degradation process was also photographed. To investigate the Cu ions mediated Fenton-like reaction, 1 mL of H_2_O_2_ aqueous solution (1 mM) was immediately added to the above solution and the MB degradation was evaluated with a similar procedure.

Additionally, using DMPO and TEMPO as spin probes, the photocatalysis mediated ROS (including ·OH, ^1^O_2_, and O_2_^·−^) generation was further detected by electron paramagnetic resonance (EPR). Typically, 0.1 mg of Cu_3_SnS_4_ NFs were added to 10 mL of deionized water under dark conditions. The suspension was then exposed to visible light irradiation and the EPR signal of the complex of ·OH, ^1^O_2_, and O_2_^·−^ with the probes was captured periodically (0, 1 and 2 min).

### The NIR mediated hyperthermia of Cu_3_SnS_4_ NFs

The NIR mediated hyperthermia and the photothermal efficiency of the Cu_3_SnS_4_ NFs were investigated using an 808 nm fiber-coupled laser system. Briefly, different concentrations of Cu_3_SnS_4_ dispersions (0, 25, 50, 100, 200 and 400 μg mL^−1^) were irradiated by an 808 nm fiber-coupled laser system for different times (0, 2, 4, 6, 8 and 10 min) at varied power density (0, 1, 1.5, 2 and 2.5 W cm^−2^). The temperature of the suspensions was recorded every 50 s with an IR camera to analyze the temperature elevation tendency. The photothermal stability of Cu_3_SnS_4_ NFs was evaluated by turning on the NIR laser (808 nm, 2.0 W cm^−2^) for 10 min and then off until the dispersions reached room temperature periodically. Nine circulations were recorded.

The photothermal conversion efficiency (η) was calculated using the following equations:

$$\eta = \frac{{hS\left( {T_{Max} - T_{Surr} } \right) - Q_{Dis} }}{{I\left( {1 - 10^{ - A808} } \right)}}$$

$$hs = \frac{{m_{D} C_{D} }}{{\tau_{s} }}$$

In these equations, *m*_*D*_ represents the solution mass and equals to 0.2 g, while *c*_*D*_ = 4.2 J g^−1^ is the heat capacity of water. *T*_*Max*_ = 42.15 °C and *T*_*Surr*_ = 25 °C are the maximum temperature of the NFs and the water during the irradiation process, respectively. *Q*_*Dis*_ is associated with the light absorbance of the solvent, which approximately equals to 0. *τ*_*s*_ is the irradiation time during the temperature rise and equals to 195.5 s. *I* is the laser power and equals to 2.0 W cm^−2^. *A*_*808*_ = 0.47 is the absorbance of the NFs at 808 nm. The photothermal conversion efficiency was thus calculated to be 55.7%.

The Cu ions released from the Cu_3_SnS_4_ NFs were investigated by ICP-OES analysis. Typically, 0.5 mg of Cu_3_SnS_4_ NFs were added to 10 mL of deionized under dark conditions at room temperature. The Cu ions release process was performed on a shaking table at a shaking speed of 180 rpm. At each time point, 1 mL of the supernate was taken out, filtrated, diluted, and measured by ICP-OES. To investigate the NIR mediated Cu ions release, the suspension was exposed to NIR irradiation (808 nm, 2.0 W cm^−2^) for 10 min.

### In vitro cytotoxicity of Cu_3_SnS_4_ NFs

Human umbilical vein endothelial cells (HUVECs) and mouse fibroblast cells (L929) were obtained from the China Center of Industrial Culture Collection. Cells were seeded separately into 96-well plates at 6 × 10^4^ cells mL^−1^. The cells were incubated in α-MEM with 10% FBS at 37 °C in 5% CO_2_ atmosphere. After 24 h, the original media was removed, and α-MEM containing a diverse concentration of Cu_3_SnS_4_ NFs (0, 10, 25, 50, 100 μg mL^−1^) was added to the plates. Cytotoxicity was detected after 24 h and 48 h co-culture using the CCK-8 method. The optical density (OD) of each solution was tested at 490 nm using an ELX-800 absorbance microplate reader.

### In vitro bacterial cell culture

Gram-negative *E. Coli* (ATCC 25,922) and Gram-positive methicillin-resistant S. aureus (MRSA, ATCC 43,300) were obtained from American Type Culture Collection (ATCC). *E. coli* (ATCC 25,922) and MRSA (ATCC 43,300) were initially seeded on solid Luria–Bertani Broth medium with streak plate method. After being cultured at 36 °C for 24 h, we collected a single bacterial colony and immerged it into 10 mL of liquid Luria–Bertani Broth medium separately for further cultivation. The centrifuge tubes were placed in a shaking incubator at 100 rpm at 30 °C for 20 h. Cells were then centrifuged (5000 rpm, 10 min, 4 °C) and washed with PBS for three times before use.

### The evaluation of antibacterial activity of Cu_3_SnS_4_ NFs in vitro

The antibacterial probability of Cu_3_SnS_4_ NFs was measured with the spread plate method. The solution was concentrated using a centrifuge (5000 rpm, 10 min, 4 °C) and the supernatant was removed. Next, the pellets were resuspended and diluted at a density of 1 × 10^5^ CFU mL^−1^ with PBS (pH 6.0) containing Cu_3_SnS_4_ NFs at 100 μg mL^−1^ and 0 μg mL^−1^, according to the optical density (OD) value tested at 650 nm. Each concentration of solution underwent various disposal: I. Control: A suspension containing 0 μg mL^−1^ of Cu_3_SnS_4_ NFs, irradiated with steady light. II. A suspension containing 0 μg mL^−1^ of Cu_3_SnS_4_ NFs, irradiated with steady light and NIR laser (808 nm, 2.0 W cm^−2^, 10 min). III. A suspension containing 100 μg mL^−1^ of Cu_3_SnS_4_ NFs, irradiated with steady light. IV: A suspension containing 100 μg mL^−1^ of Cu_3_SnS_4_ NFs, irradiated with steady light and NIR laser (808 nm, 2.0 W cm^−2^, 10 min). After the procedure, 100 μl of each diluted suspension was spread onto the solid Luria–Bertani Broth medium, incubated at 37 °C in 5% CO_2_ overnight. The number of bacterial colonies was then counted.

### In vitro fluorescence live/dead staining of bacteria

The bacterial suspensions were prepared in different methods: I. PBS. II. PBS + NIR laser (808 nm, 2.0 W cm^−2^, 10 min). III. Cu_3_SnS_4_ NFs (100 μg mL^−1^). IV. Cu_3_SnS_4_ NFs (100 μg mL^−1^) + NIR laser (808 nm, 2.0 W cm^−2^, 10 min). Before staining, the supernatant containing bacterial cells was collected and centrifuged to remove Cu_3_SnS_4_ NFs. The supernatant was also discarded to remove nucleic acids or other media components that might decrease staining efficiency. Then we resuspended the pellets with sterile water containing 0.85% NaCl, SYTO9 (10 μM) and PI (10 μM). The suspension was incubated in the dark for 15 min. The stained cells were transferred to confocal dishes. Fluorescent images of the live (green fluorescent) and dead (red fluorescent) cells were obtained using an Olympus confocal laser scanning microscope.

### Morphology observation of bacteria by bio-SEM

After the anti-bacteria test, cell morphology images were acquired by SEM scanning. The bacterial suspensions: I. PBS. II. PBS + NIR laser (808 nm, 2.0 W cm^−2^, 10 min). III. Cu_3_SnS_4_ NFs (100 μg mL^−1^). IV. Cu_3_SnS_4_ NFs (100 μg mL^−1^) + NIR laser (808 nm, 2.0 W cm^−2^, 10 min) were fixed with 2.5% glutaraldehyde overnight at 4 °C. All samples were centrifuged and resuspended with PBS (0.1 M) for four times. Then all samples were fixed with osmic acid (1%, pH 7.3) for 1 h. Before dehydration, samples were washed with PBS (0.1 M) and ultrapure water for two times. Graded alcohol (30%, 50%, 60%, 70%, 80%, 90%, 100%, 100%, and 100%) was applied to dehydrate the bacteria. During each procedure, the pellets were mixed with solution thoroughly for 10 min. All samples were dried using the supercritical CO_2_ drying technology. The bacteria morphology was visualized using bio-SEM.

### Animals

Male ICR mice aged 5 weeks were obtained from Shanghai Super—B&K laboratory animal Corp. Ltd. The Institutional Animal Care and Use Committee of Shanghai Ninth People’s Hospital, Shanghai Jiao Tong University School of Medicine approved all the animal study protocols. All animal experiments were carried out in agreement with the guidelines of Shanghai Laboratory Animal Research Center.

### In vivo wound disinfection and healing assay

The ICR mice were divided into four groups, and each group consists of four mice. All mice were raised in cages under the same condition. First, the mice were injected with 5% chloral hydrate in the abdominal cavity. After anesthesia took effect, a circular wound in 6 mm was prepared on the back of each mouse with a skin punch. One day after the wound was created, 50 μL of MRSA solution (2 × 10^8^ CFU mL^−1^) was dropped into each wound to construct the wound healing model. According to the grouping, various treatments were operated: I. PBS. II. PBS + NIR laser (808 nm, 1.0 W cm^−2^, 10 min). III. Cu_3_SnS_4_ NFs (100 μg mL^−1^). IV. Cu_3_SnS_4_ NFs (100 μg mL^−1^) + NIR laser (808 nm, 1.0 W cm^−2^, 10 min). Meantime, an IR camera was deployed to record thermal images. Body weights and wound sizes were recorded every 2 days post wounding until all mice were sacrificed on the 8th day after treatment. Meanwhile, in vivo biosafety and wound healing was investigated through HE staining, masson's trichrome staining, and immunofluorescence staining (CD31, VEGF), utilizing skin tissues and main organs (liver, spleen, lung, and kidney) harvested. For the Western blot analyses, formalin-fixed, paraffin-embedded (FFPE) skin wound samples were sectioned at a thickness of 10 μm. After sections were dewaxed by adding 500 μL of octane and vigorously shaken for 10 s, 750 μL methanol was added and shaken for another 10 s. Samples were then centrifuged (15,000 *g*, 10 min, 4 °C) to remove the upper suspension, and pellets were dried for 5 min. 50 μl of lysis buffer containing 20 mM Tris–HCl (pH 7.4) and 2% SDS was added, followed by heating at 100 °C for 20 min and 60 °C for 2 h. Samples were then collected and went through western blot assay. Expression variation of CD31 and HIF-1α was tested.

## Supplementary Information

**Additional file 1:** **Scheme S1. **Illustration of the preparation process of Cu_3_SnS_4_ NFs. **Figure S1.** HRTEM image of the Cu_3_SnS_4_ NFs. **Figure S2.** The selected area electron diffraction (SAED) pattern of the Cu_3_SnS_4_ NFs. **Figure S3.** The XPS survey spectrum of the Cu_3_SnS_4_ NFs. **Figure S4.** Influence of *t*-BuOH to the Cu_3_SnS_4_ NFs mediated methylene blue degradation. Conditions: 10 μg mL^-1^ methylene blue, 100 μg mL^-1^ Cu_3_SnS_4_ NFs, t = 120 min, 25 °C. **Figure S5. **Illustration of the process of antibacterial experiment *in vitro*. **Figure S6.** Photographs of *E. coli* and MRSA bacterial colonies formed on lysogeny broth (LB)-agar plates under visible light irradiation for different times. (B) Corresponding CFU amount of *E. coli* and MRSA bacteria after visible light irradiation for different times. **Figure S7.** (A) Photographs of *E. coli* and MRSA bacterial colonies formed on lysogeny broth (LB)-agar plates under NIR irradiation (808 nm, 10 min) with different power density. (B) Corresponding CFU amount of *E. coli* and MRSA bacteria after NIR irradiation (808 nm, 10 min) with different power density. **Figure S8.** (A) Photographs of *E. coli* and MRSA bacterial colonies formed on lysogeny broth (LB)-agar plates. I: *E. coli*, II: *E. coli* after the treatment with Cu ions extract liquid, III: MRSA IV: MRSA after the treatment with Cu ions extract liquid. (B) Corresponding CFU amount of *E. coli* and MRSA bacteria after different treatments. **Figure S9.** Relative fluorescence intensity of DCF in MRSA after different treatements. Data are presented as means ± SDs (n = 4). ^**^p < 0.01. Group I: control group in the absence of NFs; Group II: NIR group with the irradiation of NIR laser (808 nm, 1.5 W cm^-2^, 10 min) in the absence of NFs; Group III: Cu_3_SnS_4_ group with the treatment with Cu_3_SnS_4_ NFs under no laser irradiation; Group IV: Cu_3_SnS_4_ + NIR group with the treatment with Cu_3_SnS_4_ NFs plus NIR irradiation (808 nm, 1.5 W cm^-2^, 10 min). All groups were exposed to visible light for 20 min. **Figure S10.** Relative cell viability of HUVEC and L929 cells after treatement with Cu_3_SnS_4_ NFs (0-100 μg mL^-1^) for 24 h and 48 h under dark conditions. Data are presented as means ± SDs (n = 4). **Figure S11.** Time dependent change of body weight of mice after different treatments. Data are presented as means ± SDs (n = 6). **Figure S12.** *In vivo* hematological index (AST, ALT, BUN, and CREA) of male ICR mice on day 8 after different treatments. Data are presented as means ± SDs (n = 6). Group I: control group in the absence of NFs; Group II: NIR group with the irradiation of NIR laser (808 nm, 1.0 W cm^-2^, 10 min) in the absence of NFs; Group III: Cu_3_SnS_4_ group with the treatment with Cu_3_SnS_4_ NFs under no laser irradiation; Group IV: Cu_3_SnS_4_ + NIR group with the treatment with Cu_3_SnS_4_ NFs plus NIR irradiation (808 nm, 1.0 W cm^-2^, 10 min). All groups were exposed to visible light during the treatment. **Figure S13.** The major hematological indicators of healthy male ICR mice on day 8 after different treatments. Data are presented as means ± SDs (n = 6). Group I: control group in the absence of NFs; Group II: NIR group with the irradiation of NIR laser (808 nm, 1.0 W cm^-2^, 10 min) in the absence of NFs; Group III: Cu_3_SnS_4_ group with the treatment with Cu_3_SnS_4_ NFs under no laser irradiation; Group IV: Cu_3_SnS_4_ + NIR group with the treatment with Cu_3_SnS_4_ NFs plus NIR irradiation (808 nm, 1.0 W cm^-2^, 10 min). All groups were exposed to visible light during the treatment. **Figure S14.** The UV/Vis/NIR absorption spectra of the *E. coli*, DTTC and *E. coli*-DTTC-Cu_3_SnS_4_. **Figure S15.** *In vitro* SERS spectra of *E. coli*-DTTC-Cu_3_SnS_4_ under the excitation of a 785 nm laser. **Figure S16.** The concentration of Cu in the *E. coli*-DTTC-Cu_3_SnS_4_, which is positively related to the concentration of the *E. coli*.

## Data Availability

All data generated or analyzed during this study are included in this article.
