# Supplementary material for: Photogenerated reactive oxygen species and hyperthermia by Cu3SnS4 nanoflakes for advanced photocatalytic and photothermal antibacterial therapy
Source: J Nanobiotechnology. 2022 Apr 20;20:195. doi: 10.1186/s12951-022-01403-y (PMC9022271; doi:10.1186/s12951-022-01403-y)
Supplement: Supplementary file 1 — Additional file 1: Scheme S1. Illustration of the preparation process of Cu3SnS4 NFs. Figure S1. HRTEM image of the Cu3SnS4 NFs. Figure S2. The selected area electron diffraction (SAED) pattern of the Cu3SnS4 NFs. Figure S3. The XPS survey spectrum of the Cu3SnS4 NFs. Figure S4. Influence of t-BuOH to the Cu3SnS4 NFs mediated methylene blue degradation. Conditions: 10 μg mL-1 methylene blue, 100 μg mL-1 Cu3SnS4 NFs, t = 120 min, 25 °C. Figure S5. Illustration of the process of antibacterial experiment in vitro. Figure S6. Photographs of E. coli and MRSA bacterial colonies formed on lysogeny broth (LB)-agar plates under visible light irradiation for different times. (B) Corresponding CFU amount of E. coli and MRSA bacteria after visible light irradiation for different times. Figure S7. (A) Photographs of E. coli and MRSA bacterial colonies formed on lysogeny broth (LB)-agar plates under NIR irradiation (808 nm, 10 min) with different power density. (B) Corresponding CFU amount of E. coli and MRSA bacteria after NIR irradiation (808 nm, 10 min) with different power density. Figure S8. (A) Photographs of E. coli and MRSA bacterial colonies formed on lysogeny broth (LB)-agar plates. I: E. coli, II: E. coli after the treatment with Cu ions extract liquid, III: MRSA IV: MRSA after the treatment with Cu ions extract liquid. (B) Corresponding CFU amount of E. coli and MRSA bacteria after different treatments. Figure S9. Relative fluorescence intensity of DCF in MRSA after different treatements. Data are presented as means ± SDs (n = 4). **p < 0.01. Group I: control group in the absence of NFs; Group II: NIR group with the irradiation of NIR laser (808 nm, 1.5 W cm-2, 10 min) in the absence of NFs; Group III: Cu3SnS4 group with the treatment with Cu3SnS4 NFs under no laser irradiation; Group IV: Cu3SnS4 + NIR group with the treatment with Cu3SnS4 NFs plus NIR irradiation (808 nm, 1.5 W cm-2, 10 min). All groups were exposed to visible light for 20 min. Figure S10. Relative c [file 12951_2022_1403_MOESM1_ESM.docx]

Supporting Information

Photogenerated Reactive Oxygen Species and Hyperthermia by Cu_3_SnS_4_ Nanoflakes for Advanced Photocatalytic and Photothermal Antibacterial Therapy

Yangzi Yang^1,2^, Chengwei Wang^1,2^, Ning Wang^1,2^, Jiaxin Li^2,3^, Yingchun Zhu^5^, Jiantao Zai^4*^, Jingke Fu^1,2*^, and Yongqiang Hao^1,2*^

^1^Shanghai Key Laboratory of Orthopaedic Implant, Department of Orthopaedic Surgery, Shanghai Ninth People’s Hospital, Shanghai Jiao Tong University School of Medicine, Shanghai 200011, China. ^2^Clinical and Translational Research Center for 3D Printing Technology, Shanghai Ninth People’s Hospital, Shanghai Jiao Tong University School of Medicine, Shanghai 200011, China.

^3^Department of Orthopedics, The Second Affiliated Hospital of Harbin Medical University, Harbin 150081, China.

^4^Shanghai Electrochemical Energy Devices Research Center, School of Chemistry and Chemical Engineering and State Key Laboratory of Metal Matrix Composites, Shanghai Jiao Tong University, Shanghai 200240, China.

^5^Key Laboratory of Inorganic Coating Materials, Shanghai Institute of Ceramics, Chinese Academy of Sciences, Shanghai 200050, China.


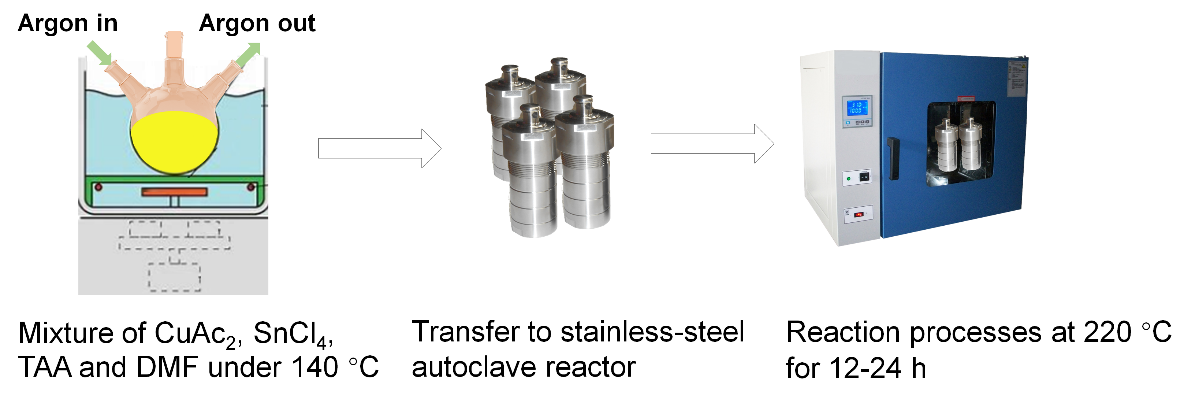


**Scheme S1.** Illustration of the preparation process of Cu_3_SnS_4_ NFs.


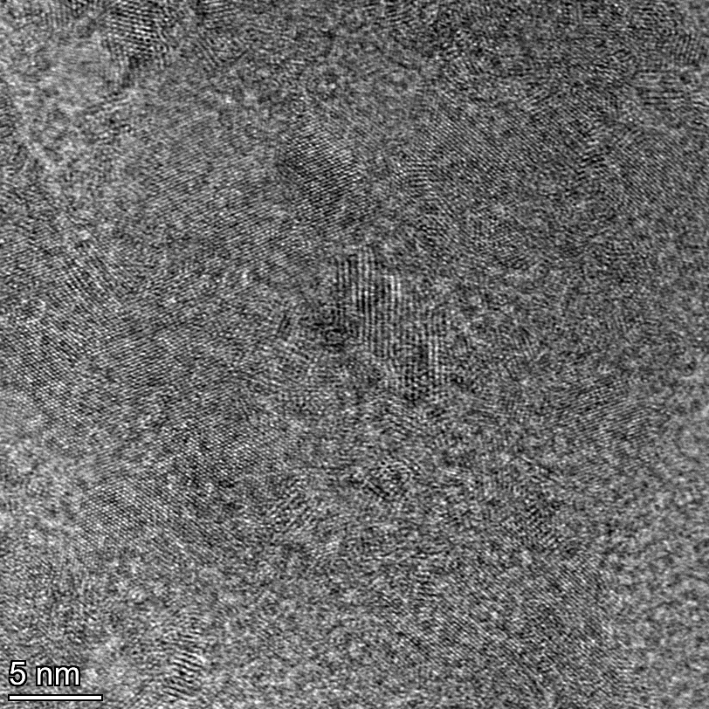


**Figure S1.** HRTEM image of the Cu_3_SnS_4_ NFs.


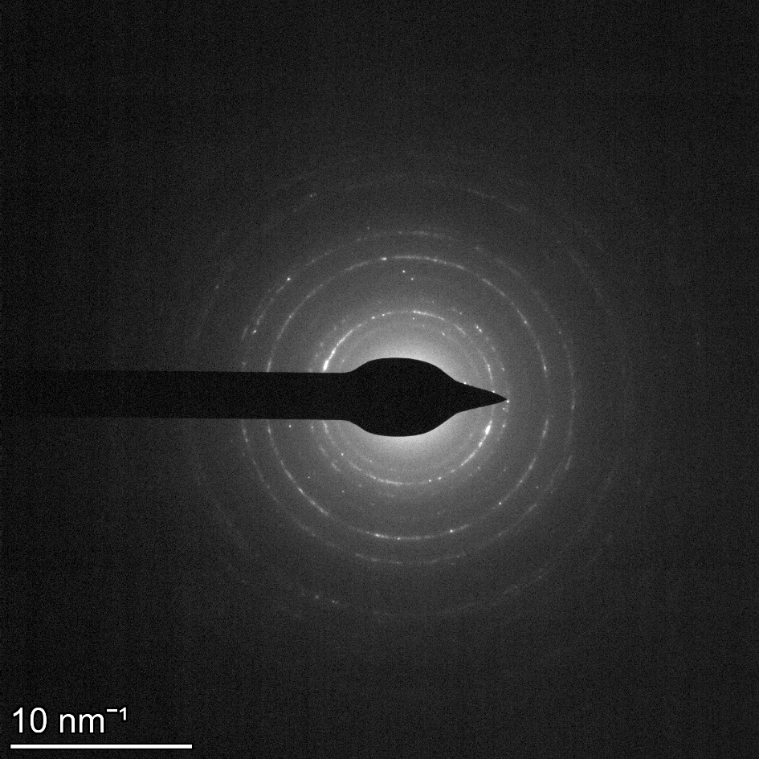


**Figure S2.** The selected area electron diffraction (SAED) pattern of the Cu_3_SnS_4_ NFs.


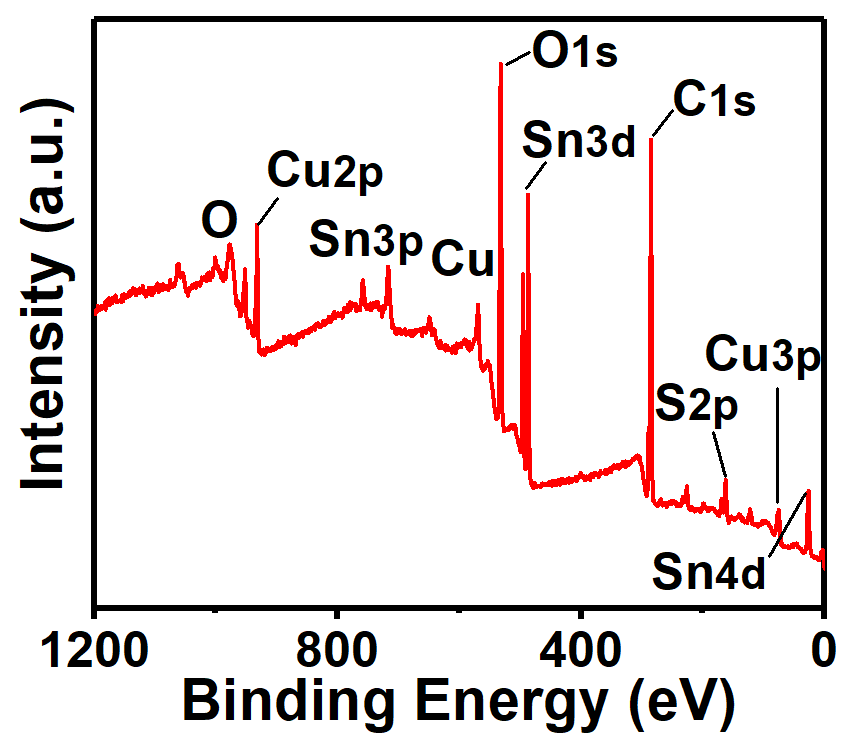


**Figure S3.** The XPS survey spectrum of the Cu_3_SnS_4_ NFs.


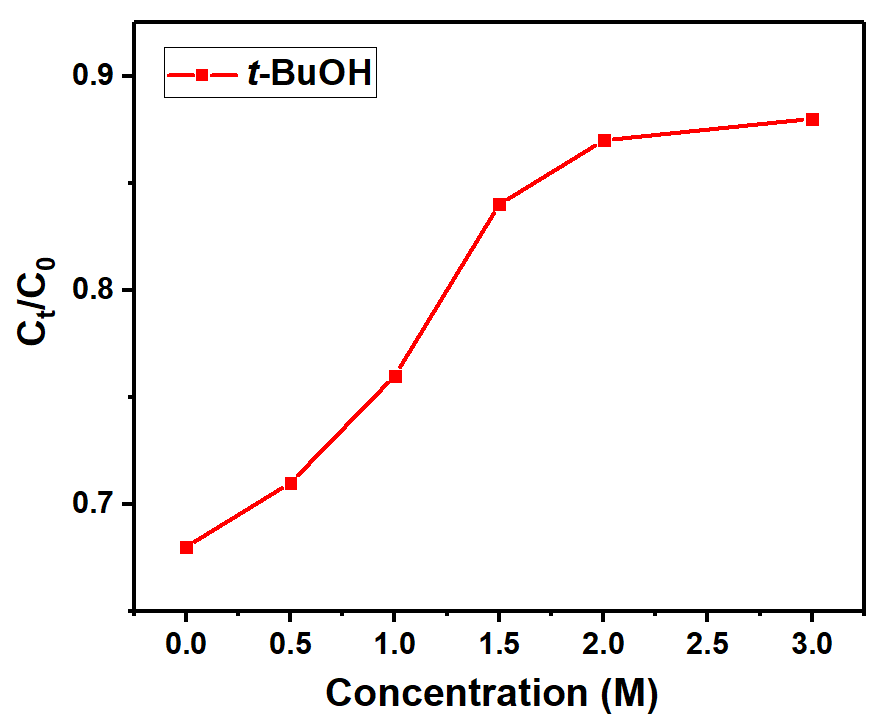


**Figure S4.** Influence of *t*-BuOH to the Cu_3_SnS_4_ NFs mediated methylene blue degradation. Conditions: 10 μg mL^-1^ methylene blue, 100 μg mL^-1^ Cu_3_SnS_4_ NFs, t = 120 min, 25 °C.


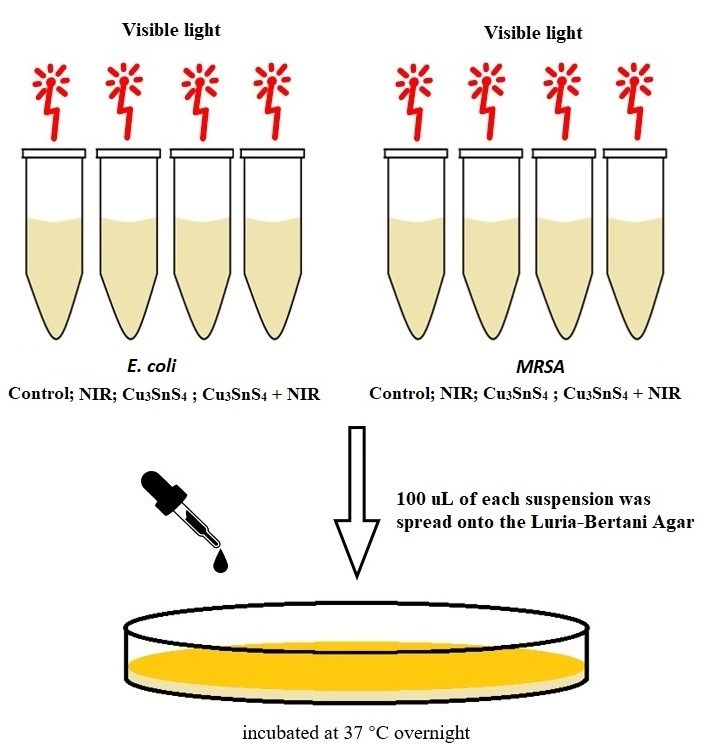


**Figure S5.** Illustration of the process of antibacterial experiment *in vitro*.


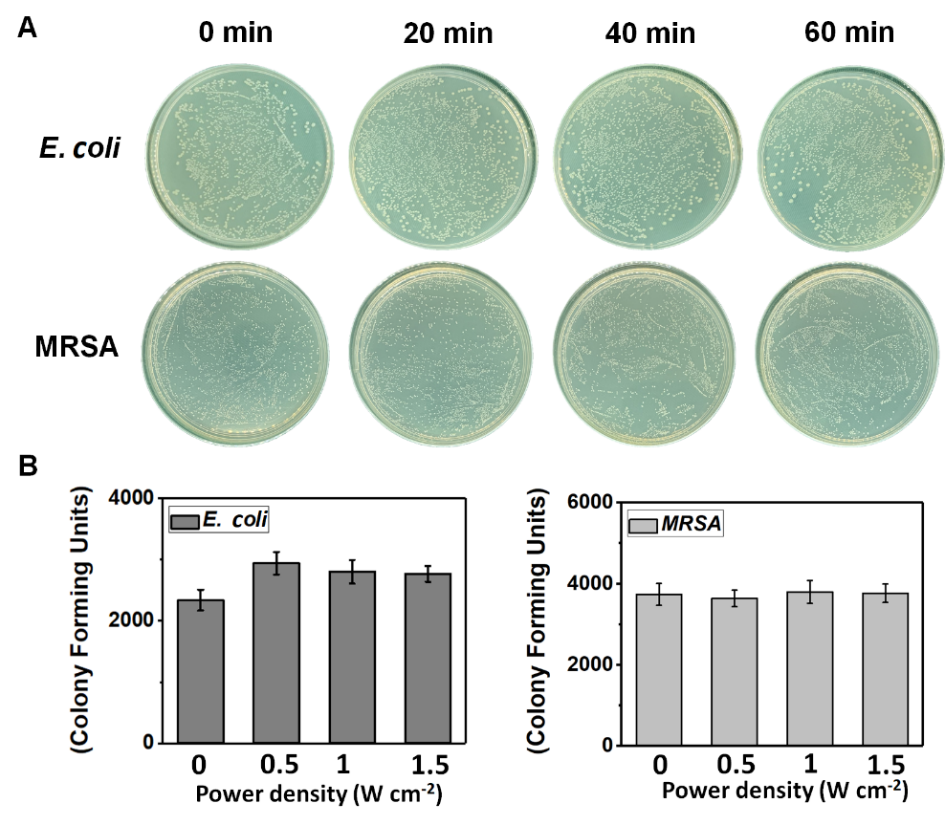


**Figure S6.** Photographs of *E. coli* and MRSA bacterial colonies formed on lysogeny broth (LB)-agar plates under visible light irradiation for different times. (B) Corresponding CFU amount of *E. coli* and MRSA bacteria after visible light irradiation for different times.


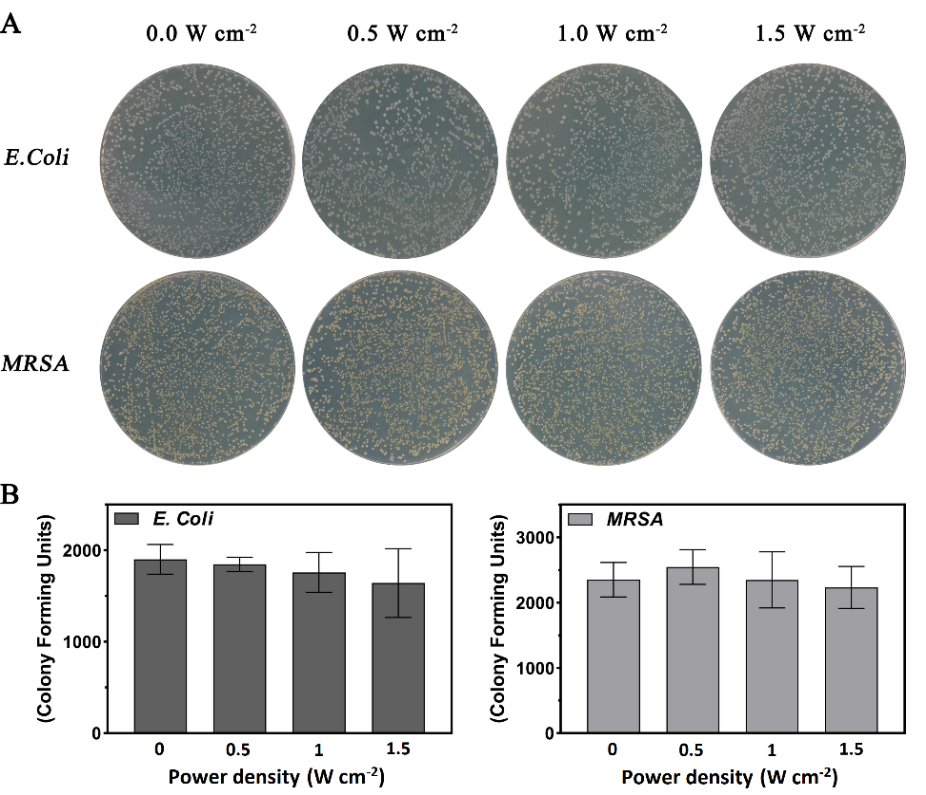


**Figure S7.** (A) Photographs of *E. coli* and MRSA bacterial colonies formed on lysogeny broth (LB)-agar plates under NIR irradiation (808 nm, 10 min) with different power density. (B) Corresponding CFU amount of *E. coli* and MRSA bacteria after NIR irradiation (808 nm, 10 min) with different power density.


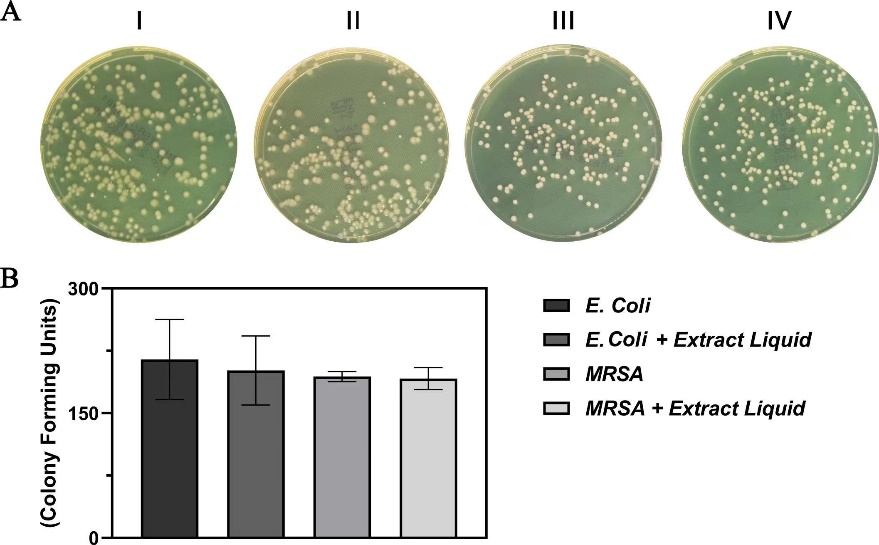


**Figure S8.** (A) Photographs of *E. coli* and MRSA bacterial colonies formed on lysogeny broth (LB)-agar plates. I: *E. coli*, II: *E. coli* after the treatment with Cu ions extract liquid, III: MRSA IV: MRSA after the treatment with Cu ions extract liquid. (B) Corresponding CFU amount of *E. coli* and MRSA bacteria after different treatments.


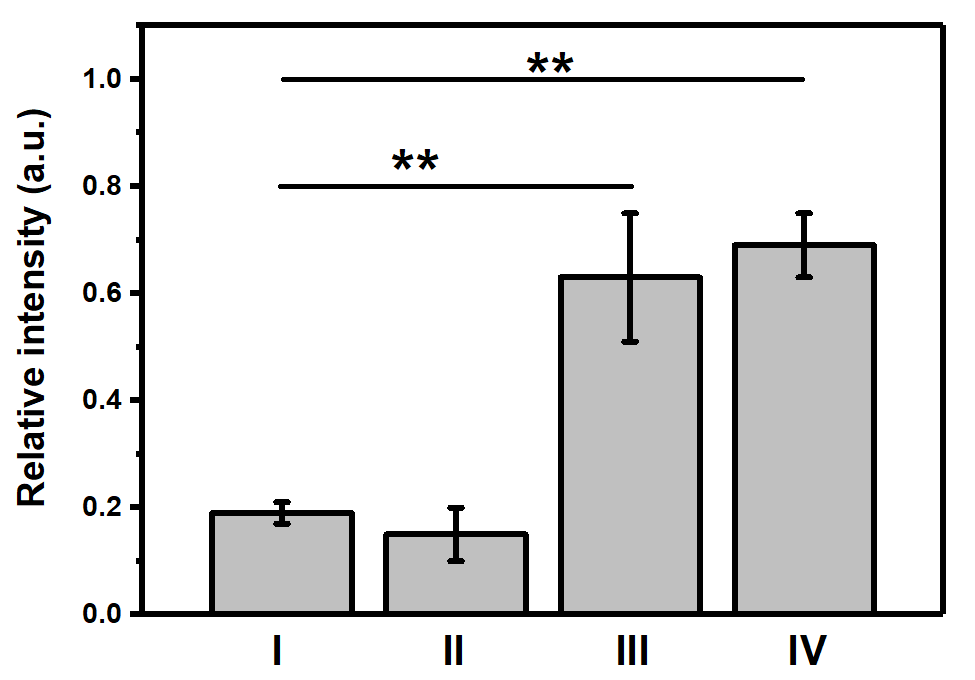


**Figure S9.** Relative fluorescence intensity of DCF in MRSA after different treatements. Data are presented as means ± SDs (n = 4). ^**^*p* < 0.01. Group I: control group in the absence of NFs; Group II: NIR group with the irradiation of NIR laser (808 nm, 1.5 W cm^-2^, 10 min) in the absence of NFs; Group III: Cu_3_SnS_4_ group with the treatment with Cu_3_SnS_4_ NFs under no laser irradiation; Group IV: Cu_3_SnS_4_ + NIR group with the treatment with Cu_3_SnS_4_ NFs plus NIR irradiation (808 nm, 1.5 W cm^-2^, 10 min). All groups were exposed to visible light for 20 min.


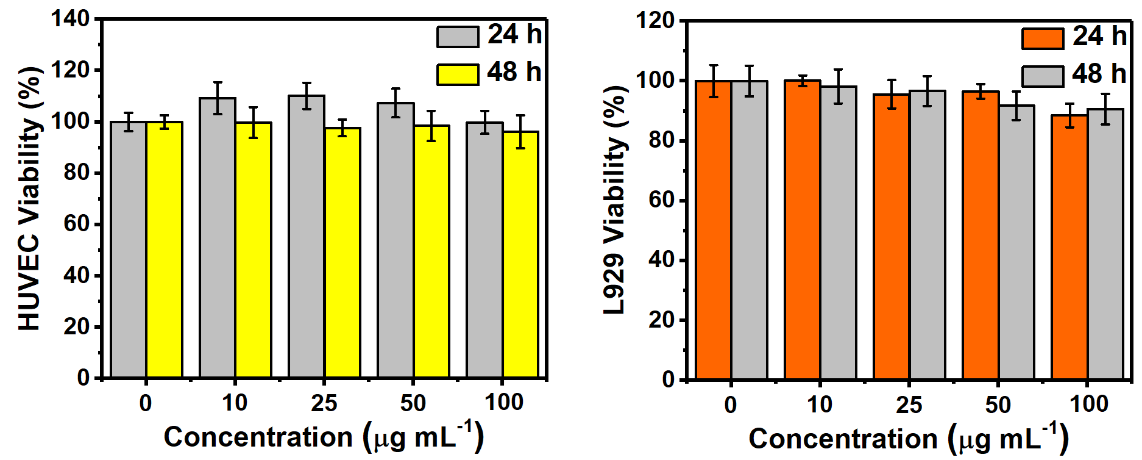


**Figure S10.** Relative cell viability of HUVEC and L929 cells after treatement with Cu_3_SnS_4_ NFs (0-100 μg mL^-1^) for 24 h and 48 h under dark conditions. Data are presented as means ± SDs (n = 4).


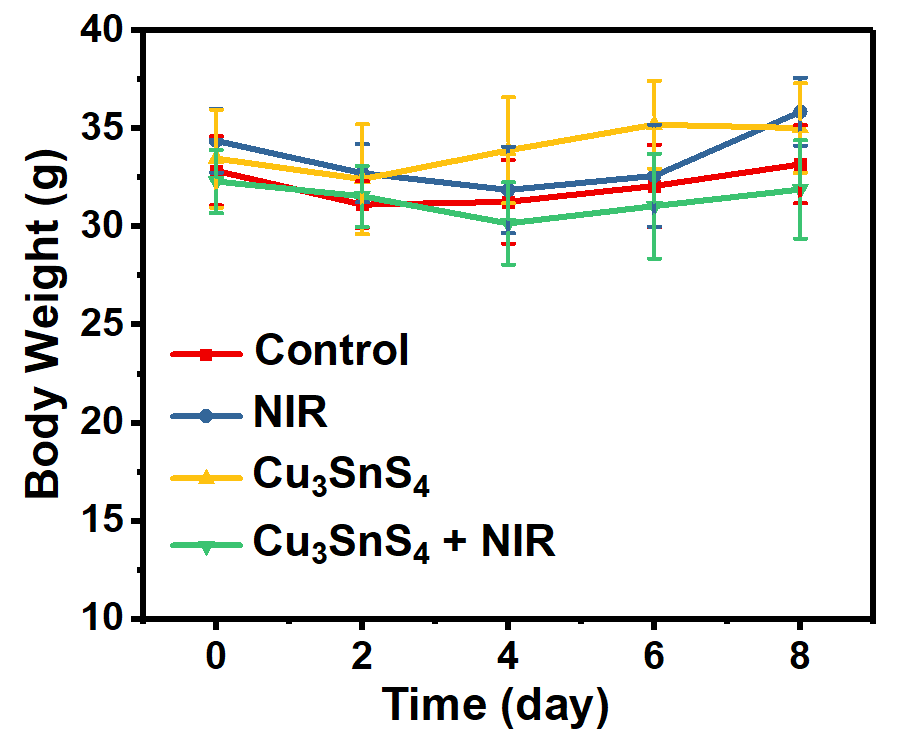


**Figure S11.** Time dependent change of body weight of mice after different treatments. Data are presented as means ± SDs (n = 6).


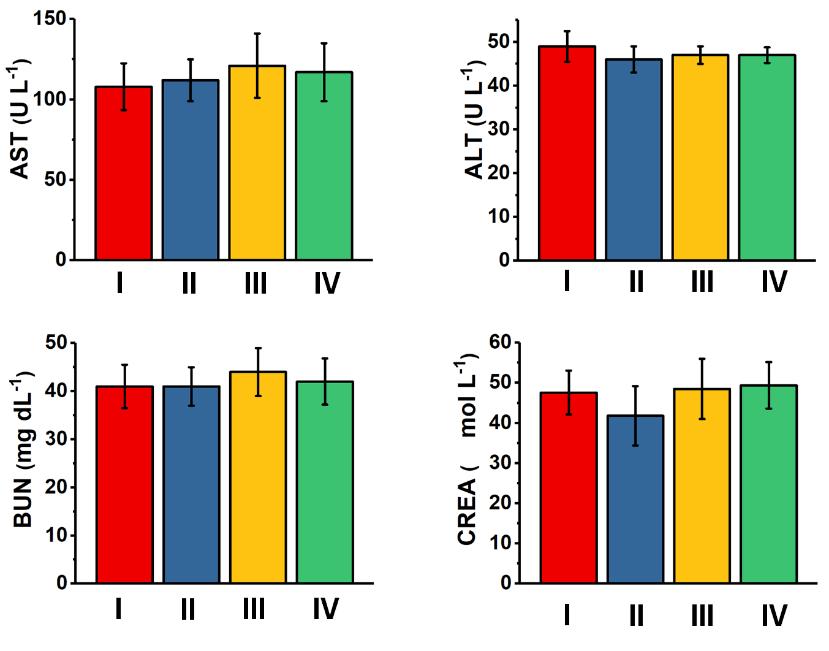


**Figure S12.** *In vivo* hematological index (AST, ALT, BUN, and CREA) of male ICR mice on day 8 after different treatments. Data are presented as means ± SDs (n = 6). Group I: control group in the absence of NFs; Group II: NIR group with the irradiation of NIR laser (808 nm, 1.0 W cm^-2^, 10 min) in the absence of NFs; Group III: Cu_3_SnS_4_ group with the treatment with Cu_3_SnS_4_ NFs under no laser irradiation; Group IV: Cu_3_SnS_4_ + NIR group with the treatment with Cu_3_SnS_4_ NFs plus NIR irradiation (808 nm, 1.0 W cm^-2^, 10 min). All groups were exposed to visible light during the treatment.


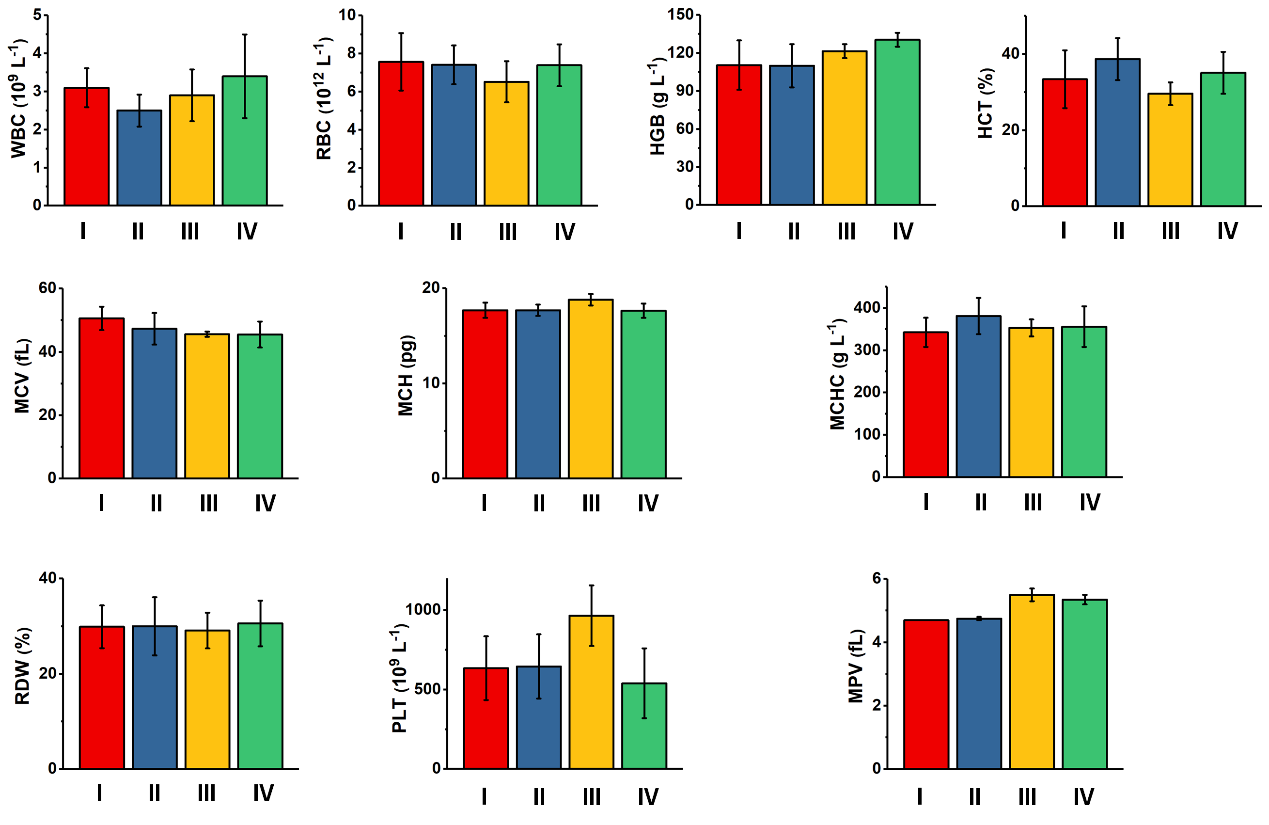


**Figure S13.** The major hematological indicators of healthy male ICR mice on day 8 after different treatments. Data are presented as means ± SDs (n = 6). Group I: control group in the absence of NFs; Group II: NIR group with the irradiation of NIR laser (808 nm, 1.0 W cm^-2^, 10 min) in the absence of NFs; Group III: Cu_3_SnS_4_ group with the treatment with Cu_3_SnS_4_ NFs under no laser irradiation; Group IV: Cu_3_SnS_4_ + NIR group with the treatment with Cu_3_SnS_4_ NFs plus NIR irradiation (808 nm, 1.0 W cm^-2^, 10 min). All groups were exposed to visible light during the treatment.


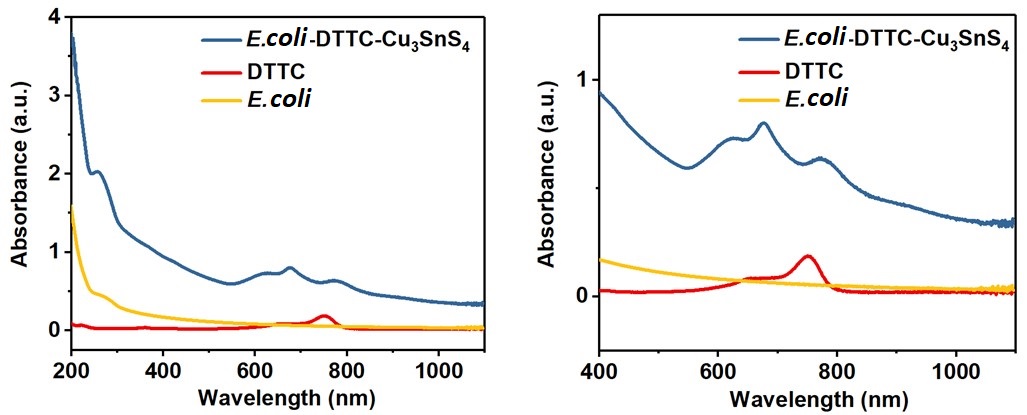


**Figure S14.** The UV/Vis/NIR absorption spectra of the *E. coli*, DTTC and *E. coli*-DTTC-Cu_3_SnS_4_.


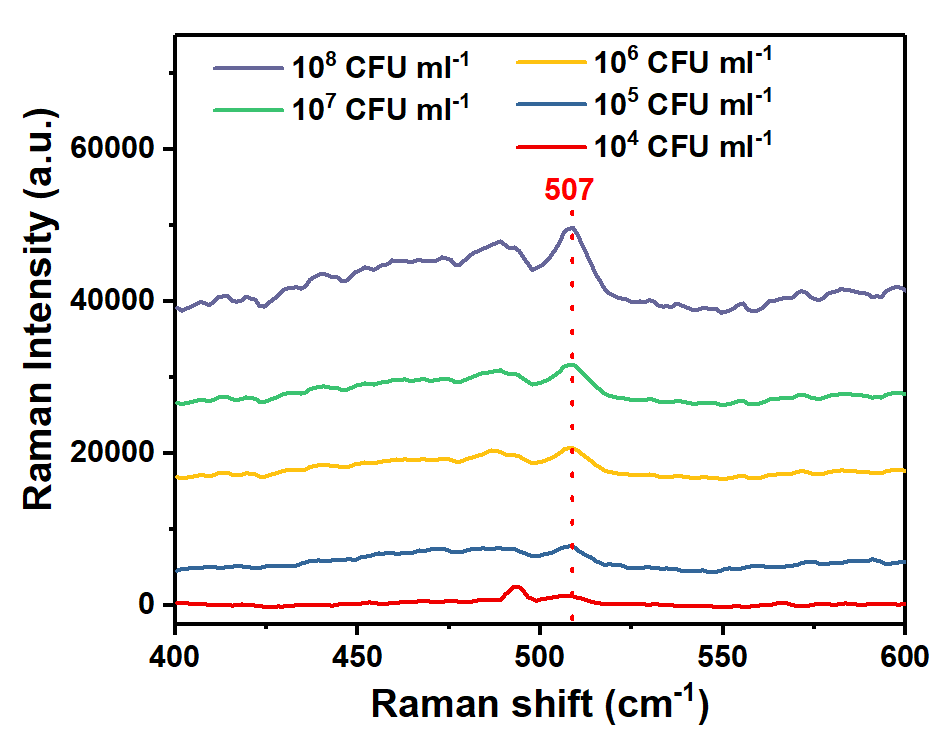


**Figure S15.** *In vitro* SERS spectra of *E. coli*-DTTC-Cu_3_SnS_4_ under the excitation of a 785 nm laser.


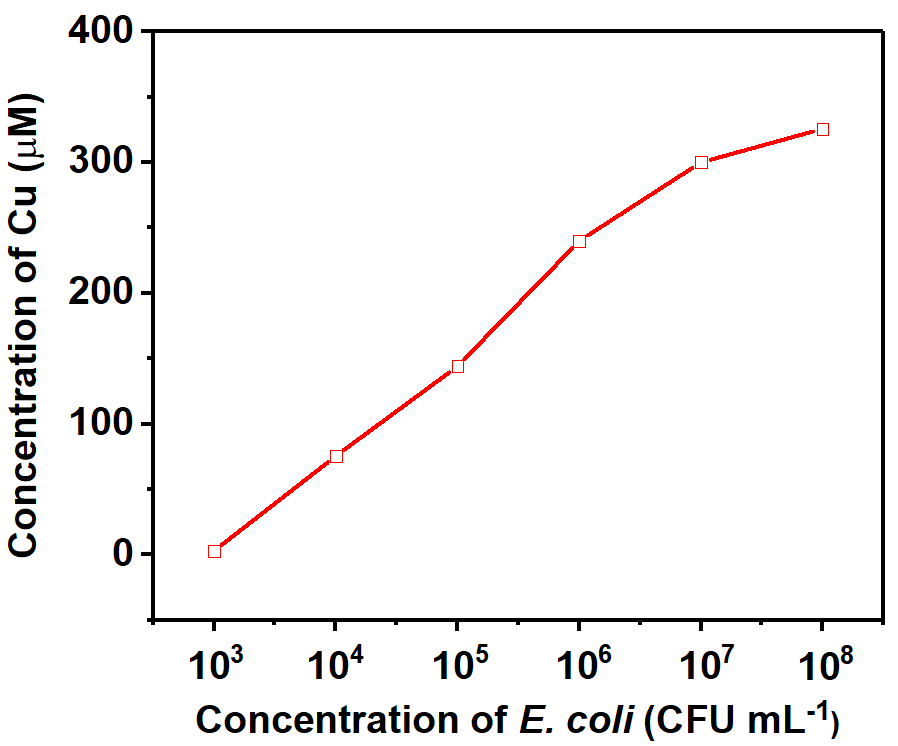


**Figure S16.** The concentration of Cu in the *E. coli*-DTTC-Cu_3_SnS_4_, which is positively related to the concentration of the *E. coli*.
